# Multifunctional Mesoporous Silica-Coated Gold Nanorods Mediate Mild Photothermal Heating-Enhanced Gene/Immunotherapy for Colorectal Cancer

**DOI:** 10.3390/pharmaceutics15030854

**Published:** 2023-03-06

**Authors:** Meirong Li, Jingyu Yang, Xinhuang Yao, Xiang Li, Zhourui Xu, Shiqi Tang, Bangxu Sun, Suxia Lin, Chengbin Yang, Jia Liu

**Affiliations:** 1 School of Biomedical Engineering, Shenzhen University Medical School, Shenzhen University, Shenzhen 518060, China; 2 Central Laboratory of The Second Affiliated Hospital, School of Medicine, The Chinese University of Hong Kong, Shenzhen & Longgang District People’s Hospital of Shenzhen, Shenzhen 518172, China; 3 Center of Assisted Reproduction and Embryology, The University of Hong Kong-Shenzhen Hospital, Shenzhen 518048, China

**Keywords:** silica-coated gold nanorods, siPD-L1, CpG ODN, gene therapy, mild photothermal therapy, immunotherapy, colorectal cancer

## Abstract

Colorectal cancer (CRC) is the third most common cancer worldwide and the second leading cause of cancer-related deaths in the world. It is urgent to search for safe and effective therapies to address the CRC crisis. The siRNA-based RNA interference targeted silencing of PD-L1 has extensive potential in CRC treatment but is limited by the lack of efficient delivery vectors. In this work, the novel cytosine-phosphate-guanine oligodeoxynucleotides (CpG ODNs)/siPD-L1 co-delivery vectors AuNRs@MS/CpG ODN@PEG-bPEI (ASCP) were successfully prepared by two-step surface modification of CpG ODNs-loading and polyethylene glycol-branched polyethyleneimine-coating around mesoporous silica-coated gold nanorods. ASCP promoted dendritic cells (DCs) maturation by delivering CpG ODNs, exhibiting excellent biosafety. Next, mild photothermal therapy (MPTT) mediated by ASCP killed tumor cells and released tumor-associated antigens, further promoting DC maturation. Furthermore, ASCP exhibited mild photothermal heating-enhanced performance as gene vectors, resulting in an increased PD-L1 gene silencing effect. Enhanced DCs maturity and enhanced PD-L1 gene silencing significantly promoted the anti-tumor immune response. Finally, the combination of MPTT and mild photothermal heating-enhanced gene/immunotherapy effectively killed MC38 cells, leading to strong inhibition of CRC. Overall, this work provided new insights into the design of mild photothermal/gene/immune synergies for tumor therapy and may contribute to translational nanomedicine for CRC treatment.

## 1. Introduction

Colorectal cancer (CRC) is a malignant tumor occurring in the colon or rectum characterized by high invasiveness, strong metastasis, easy recurrence and poor prognosis [1]. CRC is the third most common cancer and the second leading cause of cancer-related deaths in the world with an incidence of 10.2% and a mortality rate of 9.2% of all cancers [2]. So far, standard conventional treatments for CRC are chemoradiotherapy and surgical resection [3]. However, the former is accompanied by severe toxic and side effects and the latter has a high risk of anal loss [3]. Despite the emergence of total neoadjuvant therapy (TNT), limited progress has been made in improving the survival outcomes in patients with CRC [4]. Therefore, the prognosis for patients with metastatic CRC remains poor with a median 5-year survival of only 18.5–27.7% [4]. The global burden of CRC is 1.93 million new cases and 0.93 million deaths in 2020 [1]. What is worse, this burden is projected to increase to 3.2 million new cases and 1.6 million deaths by 2040 [5]. The increasing incidence and deaths make CRC a serious threat to human health and survival. It is urgent to search for safe and effective therapies to address the CRC crisis.

Programmed death-1 (PD-1) is a cell surface receptor that functions as a T cell checkpoint and plays a central role in regulating T cell exhaustion [6]. A variety of tumor cells, such as CRC, lung cancer and leukemia, can avoid the recognition and killing of T cells by overexpressing the programmed death ligand 1 (PD-L1), resulting in T cell exhaustion in the tumor microenvironment (TME) and realizing immune escape [7]. The immune checkpoint blockade (ICB) therapy, with PD-L1 as the representative therapeutic target, has achieved great success in the field of cancer immunotherapy due to its unprecedented and durable clinical response in various cancers [8,9]. However, with the emergence of drug resistance, low therapeutic response rate and cytotoxicity caused by cytokine storm, the wide application of ICBs in clinical application is limited [10]. Accordingly, developing other therapeutic methodologies to target PD-L1 for cancer treatment is urgent. Interference with PD-L1 function by various experimental methods in tumor therapy has proven that immune checkpoint silencing may be a better strategy for strengthening therapeutic efficacy than immune checkpoint blocking [11,12,13,14,15,16,17]. Blocking PD-L1 by silencing can effectively inhibit tumor growth and invasion of CRC [14,15,18,19]. RNA interference (RNAi) technology can specifically silence the expression of target genes [11,12]. siRNA-mediated RNAi, one of the most widely studied techniques in gene therapy, is considered a promising approach to treating refractory cancer due to its high specificity, rapid and robust inhibition and low toxicity [20,21,22]. Therefore, the siRNA-based RNAi-targeted silencing of PD-L1 for CRC treatment has also aroused great research enthusiasm and shown broad potential [7,19,23].

Moreover, many studies have shown that cytosine-phosphate-guanine oligodeoxynucleotides (CpG ODNs) are the most effective immune adjuvants. They can be recognized as pathogen-related molecular patterns (PAMPs) by toll-like receptor 9 (TLR9) to activate innate and acquired immune responses [24]. Thus, CpG ODNs have been used alone or in combination with other drugs for preventing or treating cancer [25,26,27]. Furthermore, CpG ODNs are excellent candidates for combination with PD-L1 blockades [24]. Immunoregulation using CpG ODNs at the tumor site could promote the maturation and antigen presentation of dendritic cells (DCs) and activate the rapid proliferation and infiltration of cytotoxic T lymphocytes (CTLs) in TME, thereby restoring resistance to the PD-L1 blockade and inhibiting the growth, metastasis and recurrence of CT26 or MC38 cell-driven CRC [27,28]. Based on the above evidence, we suggest that CpG ODNs may have great potential as immune adjuvants to improve the efficacy of siPD-L1-mediated immune checkpoint silencing in CRC. 

The therapeutic effects of either siPD-L1 or CpG ODNs largely depend on the payload and delivery efficiency of the carrier. Finding a safe and efficient co-delivery carrier of siPD-L1 and CpG ODNs is the premise of realizing their joint application. Mesoporous SiO_2_ (or Mesoporous silica, MS) nanomaterials are promising gene or drug carriers for improving cancer therapy due to their attractive properties of a high porosity and loading capacity, efficient delivery efficiency, good biocompatibility, facile surface modification and self-adjuvanticity [29,30]. Gold nanomaterials, with elegant thermal, optical or chemical properties due to quantum size effects, can be used as photothermal therapy (PTT) reagents, carriers and biosensors for therapeutic or diagnostic applications [31,32,33,34,35,36]. The nanocomposites (NPs) formed by gold nanorods (AuNRs) coated with MS possess the above advantages while functioning as carriers. MS-coated AuNR NPs could be used in siPD-L1-mediated immune checkpoint silencing for bladder cancer treatment [37] or chemotherapeutic/photothermal synergistic therapy for CRC treatment [38]. Such nanocomposites could also co-deliver doxorubicin (DOX) and Bcl-2-targeted siRNA to mediate the three-fold synergic therapy of PTT, chemotherapy and gene therapy for the potential treatment of breast cancer [39]. However, the co-delivery of CpG ODNs and siPD-L1 by MS-coated AuNR NPs for the immunological and gene synergistic therapy of CRC is still extremely rare. In addition, PTT can be divided into traditional photothermal therapy (>45 °C) and mild photothermal therapy (MPTT) (42–45 °C) [40]. MPTT could not only avoid the damage of normal tissues around the tumor caused by hyperthermia, but also increase the cell membrane permeability and improve cellular uptake and gene transfection efficiency [40,41]. Importantly, MPTT was beneficial to the survival and release of tumor-associated antigens (TAAs), it could effectively activate the antigen presentation of DCs, it could promote the proliferation and differentiation of CTLs and finally, it could effectively enhance the anti-tumor immune efficacy [40,42]. Due to the adjustability of laser power and radiation time, MS-coated AuNRs NPs have the potential to realize mild photothermal heating for promoting delivery efficiency and tumor immunotherapy efficacy. 

**Scheme 1:**
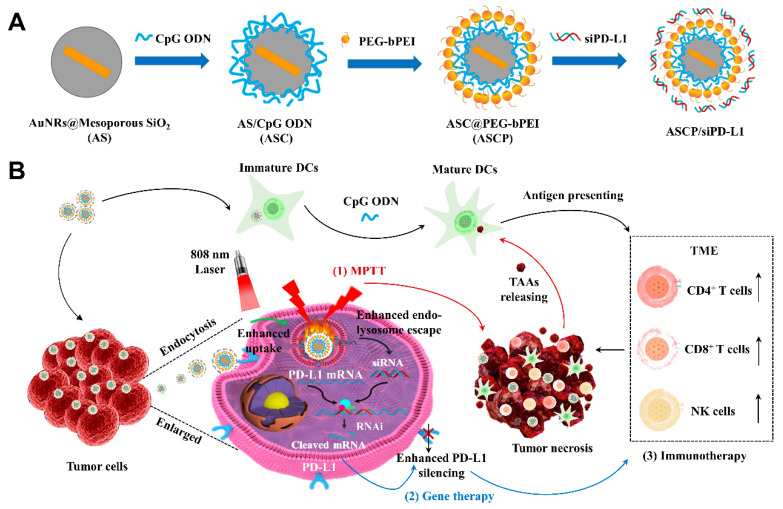
(**A**) Schematic illustration of the preparation of ASCP/siPD-L1 nanoparticles and (**B**) their application in mild photothermal heating-enhanced gene/immunotherapy for colorectal cancer. PEG-bPEI, polyethylene glycol-branched polyetherimide; DCs, dendritic cells; MPTT, mild photothermal therapy; TAAs, tumor-associated antigen; TME, tumor microenvironment. “↑” shown a significant increase in cell percentage.

In this context, we successfully constructed a novel multifunctional mesoporous silica-coated gold nanorod to mediate mild photothermal heating-enhanced gene/immunotherapy for CRC. As illustrated in Scheme 1, AuNRs@MS (AS) NPs were synthesized by coating the mesoporous silica (MS) shells with gold nanorods (AuNRs) as the cores. Then, the co-delivery vector AuNRs@MS/CpG ODN@PEG-bPEI NPs (ASCP) were prepared by two-step surface modification of CpG ODNs-loading and polyethylene glycol-branched polyethyleneimine (PEG-bPEI)-coating. Exhibiting excellent biosafety in vitro, ASCP-based NPs promoted the maturity and antigen presentation ability of DCs by delivering CpG ODNs. Next, ASCP-based NPs not only mediated mild photothermal heating to kill tumor cells, but also facilitated the release of tumor-related antigens (TTAs) from tumor lysates to promote the DCs maturity and antigen presentation further. Furthermore, the transfection and endo-lysosomal escape of payloads were strengthened by mild photothermal heating mediated by ASCP-based NPs, resulting in an enhanced PD-L1 gene silencing effect to relieve T cell exhaustion and inhibit tumor immune escape. The enhanced DCs maturity and PD-L1 gene silencing effectively promoted the proliferation and infiltration of effector T lymphocytes and natural killer cells (NKs) in TME, which could further effectively kill tumor cells following the tumor ablation effect of PTT, and ultimately lead to the strong inhibition of CRC. The findings in this study provide new insights into the design of gene/immune/mild photothermal synergies for tumor therapy and may contribute to translational nanomedicine for CRC treatment.

## 2. Materials and Methods

### 2.1. Materials

Cetyltrimethylammonium bromide (CTAB, No. H9151), polyethylene glycol-branched polyetherimide (PEG-bPEI, No. 900926), ribonuclease A (RNase A, No. 10109169001) and 4’,6-diamidino-2-phenylindole (DAPI, MBD0015) were purchased from Sigma-Aldrich Co., Ltd. (St. Louis, MO, USA). Gold chloride solution (HAuCl_4_, No. g109456), NaBH_4_ (No. s108355), silver nitrate solution (AgNO3, No. s116264), sodium hydroxide solution (NaOH, No. S817971), formaldehyde solution (37%, No. F809702), ethyl acetate (EA, No. E809178), L-ascorbic acid (No. A800296) and tetraethyl orthosilicate (TEOS, No. T819507) were obtained from Macklin Co., Ltd. (Shanghai, China). Phosphate buffer solution (PBS, No. P1010), tris-acetate-EDTA buffer (TAE, No. T1060) and 0.25% trypsin-EDTA solution (No. T1300) were purchased from Solarbio Biotech (Shanghai, China). Hoechst 33342 (0.01 mg/mL; No. 62249), Roswell Park Memorial Institute 1640 medium (RPMI 1640, No. 11875119), penicillin-streptomycin solution (PS, No. 15070063), fetal bovine serum (FBS, NO.10100147) and Lipofectamine™ 3000 (Lipo3000, No. L3000001) were purchased from Gibco Biotech (ThermoFisher Scientific, Waltham, MA, USA). Lyso-tracker red (DND 99, No. C1046), calcein-AM (No. C2012) and red blood cell lysate (No. C3702) were purchased from Beyotime Biotech (Shanghai, China). Cell Counting Kit-8 (CCK-8, No. BS350A), granulocyte-macrophage colony-stimulating factor (GM-CSF, No. BSEM-026), interleukin-4 (IL-4, No. BSEM-004) and lipopolysaccharide (LPS, No. BS904) were from Biosharp Co., Ltd. (Beijing, China). TRIcom reagent (No. TR251) was from TIANMO BIOTECH Co., Ltd. (Beijing, China). QuantiTect Reverse Transcription Kit (No. 205311) was purchased from QIAGEN Biology Co., Ltd. (Frankfurt, Germany). Bestar SybrGreen qPCR Master Mix Kit (No. DBI-2043) was from DBI Bioscience Co., Ltd. (Ludwigshafen, Germany). 5-(and 6-)carboxyfluorescein diacetate succinimidyl ester (CFSE or CFDA-SE, No. abs9106) and propyl iodide solution (PI, No. abs9358) were purchased from Absin Biology (Shanghai, China). All the flow cytometry antibodies of Ms CD45 PerCP 30-F11 (No. 557235), Ms CD3e APC 145-2C11 (No. 553066), Ms CD4 PE-Cy7 RM4-5 (No. 552775), Ms CD8a FITC 53-6.7 (No. 553030), Ms CD49b PE HM Alp2 (No. 558759), Ms CD11c APC HL3 (No. 550261), Ms CD86 PE GL1 (No. 561963) and Ms I-A I-E Alexa 488 M5/114.15.2 (No. 562352) were purchased from BD Pharmingen (Franklin Lake, NJ, USA).

### 2.2. Synthesis of siRNAs and Primers

All the custom synthesized siRNAs were purchased from Shanghai GenePharma Co., Ltd. (Shanghai, China) and all the custom synthesized primers and CpG ODN 2395 (abbreviated as CpG) were purchased from Sangon Biotech (Shanghai, China). 

The siRNA sequences were listed as follows: sense of siPD-L1: 5′-GACUCAAGAUGGAACCUGAdTdT-3′, antisense of siPD-L1:5′-UCAGGUUCCAUCUUGAGUCdTdT-3′, sense of siNC: 5′-CGAAGUGUGUGUGUGUGGCdTdT-3′, antisense of siNC: 5′-GCCACACACACACACUUCGdTdT-3′. siNC: negative control siRNA. The sequence of CpG was 5′-TCGTCGTTTTCGGCGCGCGCCG-3′. The siRNA labeled with Cy5 (siCy5) or FAM (siFAM) in the 5’ tag and CpG labeled with Cy3 (CpG^Cy3^) in the 5’ tag was used for fluorescence detection. The primer sequences for qPCR were listed as follows: Mouse-PD-L1-F: 5′-TGCTGCATAATCAGCTACGG-3′, Mouse-PD-L1-R: 5′-CCACGGAAATTCTCTGGTTG-3′. Mouse-GAPDH-F: 5′-GTGGACCTCATGGCCTACAT-3′, Mouse-GAPDH-R: 5′-TGTGAGGGAGATGCTCAGTG-3′.

### 2.3. Preparation of AS NPs

The silica-coated gold cores with a shell-core structure were prepared by a method previously reported in the literature [39,43,44]. Typically, the gold seed solution was prepared by mixing aqueous HAuCl_4_ (0.01 M, 0.25 mL) and cetyltrimethylammonium bromide (CTAB, 0.1 M, 10 mL) in a 15 mL plastic tube. A freshly prepared, ice-cold NaBH_4_ solution (0.01 M, 0.6 mL) was then injected quickly into the mixture solution, followed by rapid inversion for 2 min. The seed solution was kept at room temperature (RT) for at least 2 h before use. To grow gold nanorods (AuNRs), HAuCl_4_ (0.01 M, 2.0 mL) and AgNO_3_ (0.01 M, 0.4 mL) were mixed with CTAB (0.1 M, 40 mL) in a 50 mL plastic tube. HCl (1.0 M, 0.8 mL) was then added to adjust the pH of the solution to 1–2, followed by the addition of ascorbic acid (0.1 M, 0.32 mL). Finally, the seed solution (0.096 mL) was injected into the growth solution. The solution was gently mixed for 10 s and left undisturbed at RT for at least 6 h before use. Notably, the amount of AuNRs before and after should be quantified. For further preparation of AuNRs@MS NPs (AuNRs NPs coated with mesoporous silica shells), the CTAB was subsequently removed by centrifugation, the pH was adjusted to 10 by a NaOH solution (0.1 M), TEOS/ethanol solution was added to the mixture in three portions and the reaction was gently stirred for 48 h at RT to obtain AuNRs@MS NPs (abbreviated as AS NPs) with a shell-core structure.

### 2.4. Modification from AS NPs to ASCP NPs

The AS NPs solution and CpG solution were mixed at the AS:CpG mass ratio of 5:1. Next, the mixture was stirred with a magnetic mixer at RT, 100 rpm for 6 h. The excess CpG was removed three times by repeated operations of 5 min-centrifugation (10,000 rpm, 25 °C) and pure water washing. In order to get the siRNA-load capacity, PEG-bPEI was modified in the outer layer of the ASC NPs. Briefly, 1 mg of AS NPs was dissolved in 10 mL of ultra-pure water, then mixed with 0.05 mL of PEG-bPEI solution (100 mg/mL). Then, the mixture was magnetically stirred overnight at RT, 300 rpm. After the three repeated operations of 10 min-centrifugation (10,000 rpm, 25 °C) and pure water washing, the excess PEG-bPEI was removed. Finally, the CpG ODN/siRNA co-delivery vectors of ASCP were successfully obtained.

### 2.5. Preparation of ASCP/siRNA NPs

ASCP NPs and siRNA were dissolved in diethylpyrocarbonate (DEPC) water. ASCP/siRNA NPs were prepared by gently mixing the ASCP solution with the siRNA solution at different mass ratios (3.75:1, 7.5:1, 15:1, 22.5:1, 30:1) and incubated at RT for at least 40 min. All the weights of ASCP-based NPs were calculated by gold nanorod cores. The dose of siRNA was fixed at 0.133 μg (10 pmol) in each sample well. Then the siRNA binding capacity of ASCP NPs was evaluated by the agarose gel electrophoresis (110 V, 10 min) in a tris-acetate-EDTA (TAE) running buffer. The gel was imaged under UV transillumination (Fluor Chem E, Protein Simple, San Jose, CA, USA) and the gray values of the gel images were analyzed by Image J software v.1.53 (National Institutes of Health, NIH, Bethesda, MD, USA).

### 2.6. Characterization

The zeta potential (surface potential) values and hydrodynamic diameters of these NPs with different degrees of modification (AS NPs, ASC NPs, ASCP NPs and ASCP/siRNA NPs) were measured by dynamic light scattering (DLS, Nano-ZS90, Malvern, UK) and their morphological properties were detected by transmission electron microscopy (TEM, HT7700, Hitach Ltd., Ibaraki, Japan). The absorbance intensity of the ASC aqueous solution was measured at 275 nm by a UV-Vis-NIR spectrophotometer (TP-720, Tuopu Instrument, Tianjin, China). The plasmonic absorptions of AuNRs before and after coating with MS shells were also measured using the UV-Vis-NIR spectrophotometer. The stability of ASCP NPs (2 μg/mL) before and after irradiation (808 nm laser, 1 W/cm^2^) was further investigated by TEM.

### 2.7. In Vitro Photothermal Properties Measurement

For the concentration-dependent evaluation of photothermal properties in vitro, 1 mL aqueous dispersion of ASCP NPs with different concentrations (1, 2, 4, 8 μg/mL) was irradiated by an 808 nm laser (1 W/cm^2^) for 10 min. For power density-dependent evaluation, a 1 mL aqueous dispersion of ASCP (2 μg/mL) was irradiation by an 808 nm laser at various laser densities for 10 min. For the photothermal stability measurement, a 1 mL aqueous dispersion of ASCP (2 μg/mL) was irradiated in four consecutive laser-on/off cycles with a power density of 0.5 W/cm^2^. During the observation of irradiation, the real-time temperature images and temperature alteration with an accuracy of 0.1 °C were recorded by a FLIR A300 infrared thermal imaging camera. 

### 2.8. Cells and Culture 

The mouse colorectal cancer cell line MC38 and mouse breast cancer cell line 4T1 were purchased from the National Infrastructure of Cell Line Resource (Beijing, China). After the mice were sacrificed with an overdose of isoflurane, the spleen was isolated, cut up, gently ground and filtered through a 200-mesh screen to obtain the single-cell suspension of the spleen cell. Meanwhile, the tibia, femur and ilium of the lower extremities in mice were isolated. Then a 1 mL syringe was used for flushing out bone marrow cells (BMCs) from the marrow cavity of these bones with RPMI 1640 complete medium. After 2 min mild treatment with red blood cell lysate and washing with PBS, experimental splenocytes and BMCs were obtained, respectively. All the cells were cultured in RPMI 1640 complete medium containing 10% FBS and 1% PS, and maintained in a 37 °C incubator with 5% CO_2_.

### 2.9. Mice and Feeding 

Six–eight weeks old female C57BL/6J mice were purchased from the Medical Laboratory Animal Center of Guangdong Province, China. The mice were kept in an individually ventilated cages (IVCs) system in a specific pathogen freedom (SPF) animal house. The SPF environment conditions were set to a temperature of 22.5 ± 2.5 °C, humidity of 55 ± 15%, noise of less than 60 decibels, accompanied by a natural 12-h cycle of alternating light and dark. All the animal experiments were approved by the Animal Ethical and Welfare Committee of Shenzhen University (AEWC) and were performed strictly according to animal care guidelines. 

### 2.10. Hemolysis Assay

The hemolysis assay was performed to evaluate the biocompatibility of ASCP NPs on red blood cells (RBCs). An amount of 1 mL of blood from healthy C57BL/6J mice was diluted in a 9 mL phosphate-buffered solution (PBS, pH 7.4). Then, the blood diluent was centrifuged at 4 °C, 5000 rpm for 10 min and the supernatant was discarded. After repeating the above operation three times, the precipitation of RBCs was diluted in 1 mL PBS. Finally, the RBCs working solution for the hemolysis assay consisted of 5% (*v*/*v*) of RBCs in PBS. To evaluate the hemolytic effects, 200 μL of RBCs working solution were incubated with 800 μL of ASCP NPs with different concentrations for 4 h in a 37 °C incubator. An equal volume of incubated H_2_O or PBS was used as a positive control or negative control (NC), respectively. After incubation, the samples were centrifugated at 5000 rpm for 5 min at RT. In addition, 100 μL of supernatants were extracted to quantify hemoglobin by recording the absorbance at 540 nm (*A*_540_) with a microplate reader (EPOCH-2, Bio-Tek Instruments, Winooski, VT, USA). The percentage of the hemolysis rate was calculated as follows: hemolysis rate (%) = (*A*_540_ of sample − *A*_540_ of NC) / (*A*_540_ of PC − *A*_540_ of NC) × 100%.

### 2.11. In Vitro Cell Cytotoxicity

Cell viability was measured by utilizing the Cell Counting Kit-8 (CCK-8) assay. Different cells (MC38, 4T1 and splenocytes) were seeded into 96-well plates at a density of 1 × 10^4^ cells/well and incubated overnight, respectively. Subsequently, cells were incubated in 100 μL RPMI 1640 complete medium with different concentrations of ASCP NPs (0, 0.25, 0.5, 1, 2, 4, 8, 16 μg/mL) for 24 h. ASCP NPs-free treated cells were used as the control group (Blk group). After adding 10 μL CCK-8 in each well, cells were incubated for 2 h at 37 °C. Then, the absorbance was measured with a microplate reader (EPOCH-2, Bio-Tek Instruments, Winooski, VT, USA) at a wavelength of 450 nm. The cell viability was calculated according to the following formula: cell viability (%) = *A*_450_ of the test well/*A*_450_ of the control well (Blk group) × 100%. 

### 2.12. Preparation of BMDCs

Mouse bone marrow cells (BMCs) were isolated and co-induced to prepare bone marrow-derived dendritic cells (BMDCs) using interleukin-4 (IL-4) and granulocyte macrophage colony-stimulating factor (GM-CSF) according to the method reported by Tang et al. [45]. In brief, mouse BMCs were seeded in a 24-well plate at a density of 1 × 10^5^ cells per well and cultured in 1 mL of RPMI 1640 complete medium. GM-CSF (20 ng/mL) and IL-4 (10 ng/mL) were added into the cultures on day 1 and day 3, respectively, to promote the differentiation of BMCs into BMDCs. After observing most cells sticking to the wall on day 8, the treated BMCs were digested by trypsin, washed with PBS and then stained with the antibody of Ms CD11c APC HL3. Finally, the expression of CD11c^+^ in the cells was detected by flow cytometry (FCM; FACS Aria II, BD medical device, Franklin Lake, NJ, USA) to confirm whether the preparation of BMDCs was successful.

### 2.13. Transfection Efficiency of ASCP/siRNA NPs

MC38 cells were seeded in a 24-well plate at a density of 1 × 10^5^ cells per well and cultured in 1 mL of RPMI 1640 complete medium overnight for adherent. After removing the complete medium, cells were washed twice with 1 mL of PBS and 0.5 mL of serum-free RPMI 1640 medium was added to each well. Meanwhile, the ASCP NPs and siRNA labeled with FAM (siFAM) were mixed and incubated at the weight ratio of 7.5:1 at RT for at least 40 min to form ASCP/siFAM NPs for transfection. The final transfected concentration of ASCP NPs was 2 μg/mL and the dose of siRNA was 0.3253 μg (25 pmol). The Lipo3000 was used as the positive control and PBS was used as the negative control. Afterward, cells were transfected with the ASCP/siRNA NPs for 4 h in a 37 °C incubator with 5% CO_2_. Then, cells were treated with or without a 10 min-irradiation inspired by an 808 nm laser at the power density of 0.5 W/cm^2^ and cultured for another 4 h. After transfection, the old medium was discarded, the cells were washed twice with 1 mL PBS and 1 mL of RPMI 1640 complete medium was added for further culturing. Next, the 8 h post-transfection cells were washed three times with PBS and collected after trypsinization with a 0.25% Trypsin-EDTA solution. Finally, flow cytometry (FCM; CytoFLEX, Beckman, CA USA) was used to detect the intracellular transfection efficiency of FAM-labeled-ASCP/siRNA NPs with the channel set as FAM. Meanwhile, the mean fluorescence intensity (MFI) of the FAM channel recorded in FCM detection was also quantified.

### 2.14. Intracellular Uptake

To explore the co-delivery ability of ASCP to CpG and siRNA in MC38 cells, siRNA labeled with FAM (siFAM) and CpG labeled with or without Cy3 were used in the preparation process of ASCP-based NPs from the previous method. Firstly, the ASCP NPs or ASCP^Cy3^ NPs and siFAM were mixed and incubated at a mass ratio of 7.5:1 at RT for 40 min to form ASCP/siFAM NPs and ASCP^Cy3^/siFAM NPs. Subsequently, MC38 cells seeded in confocal dishes (5 × 10^5^ cells/dish) were transfected with the NPs of ASCP^Cy3^, ASCP/siFAM and ASCP^Cy3^/siFAM. The final transfected concentration of ASCP NPs was 2 μg/mL and the dose of siRNA was 50 pmol. After 6 h-transfection, cells were washed three times with 1 mL PBS and the nuclei were stained with 500 μL of Hoechst 33342 (0.01 mg/mL) for 15 min at 37 °C. Next, cells were washed with 1 mL PBS three times after removing the staining buffer. Finally, cells were soaked in 1 mL PBS for fluorescence imaging by using a confocal laser scanning microscope (CLSM; ZEISS LSM880 AiryScan, Jena, Germany) with the filters set for DAPI, FAM and Cy3.

For the intracellular uptake test of NPs by BMDCs, the prepared BMDCs were seeded in confocal dishes (5 × 10^5^ cells/dish) and cultured overnight for adherence. Then, BMDCs were transfected with ASCP^Cy3^ NPs and ASCP^Cy3^/siFAM NPs, same as the above methods. After the 6 h transfection, BMDCs were treated with three repeated PBS washings, followed by 30-min-staining of DAPI (0.01 mg/mL). The BMDCs were then washed with PBS 3 times and 1 mL of PBS was added for confocal fluorescence detection. Images were taken by the CLSM with the filters set for DAPI, FAM and Cy3 to assess the cellular uptake of ASCP-based NPs by BMDCs.

### 2.15. Endo-Lysosome Escape Observation

MC38 cells seeded in confocal dishes (5 × 10^5^ cells/dish) were transfected with ASCP/siFAM NPs prepared at a weight ratio of 7.5:1 for 4 h. The final transfected concentration of ASCP NPs was 2 μg/mL and the dose of siRNA was 50 pmol. Next, cells were irradiated with or without an 808 nm laser (0.5 W/cm^2^) for 10 min and cultured for another 4 h. After a total 8-h transfection, cells were washed three times with PBS. Then, the cells were stained with 1 mL PBS containing 50 nM of Lysotracker Red and 1 μg/mL Hoechst 33342 for 30 min. Next, the staining buffer was removed and the cells were washed three times with PBS. Finally, the cells were re-immersed in 1 mL PBS for endo-lysosome escape observation under the above CLSM with the filters set for DAPI, FAM and Cy3.

### 2.16. In Vitro Immune Activation Effect Induced by ASCP-Based NPs Only

The prepared BMDCs and splenocytes were seeded in a 24-well plate at a density of 1 × 10^5^ cells per well and cultured overnight. Then PBS, CpG (1 mg/mL), lipopolysaccharides (LPS, 1 mg/mL), ASCP NPs (2 μg/mL) and ASCP/siPD-L1 NPs (2 μg/mL by ASCP NPs mass) were added to different culture wells of cells for 24-h-incubation. Then, cells were digested by trypsin and washed with PBS for three times. For the activation effect test of ASCP-based NPs on BMDCs, the treated BMDCs were stained with the antibodies of Ms CD45 PerCP 30-F11, Ms CD11c APC HL3, Ms CD86 PE GL1 and Ms I-A I-E Alexa 488 M5/114.15.2 (dye for MHC-II^+^ cells). Finally, the maturity of BMDCs gated in CD45^+^CD11c^+^ cells was measured by flow cytometry to assess the in vitro immune activation effect induced by co-incubation on BMDCs. The CD86^+^MHC-II^+^ cells were mature BMDCs. For the activation effect test of ASCP-based NPs on splenic lymphocytes, the treated splenocytes were stained with the antibodies of Ms CD45 PerCP 30-F11, Ms CD3e APC 145-2C11, Ms CD4 PE-Cy7 RM4-5, Ms CD8a FITC 53-6.7 and Ms CD49b PE HM Alp2. Finally, the activation effect of different formulations of NPs on the splenic lymphocyte differentiation was detected by flow cytometry gated in CD45^+^ cells. CD3 was the marker of T lymphocytes, CD4 and CD8 were the markers of effector T lymphocytes and CD49b was the marker of natural killer cells (NKs). 

### 2.17. Fluorescence Images of Live–Dead Staining

MC38 cells were seeded into a 24-well plate (5 × 10^4^ cells/well) and incubated overnight for adherence. After removing the complete medium, cells were washed twice with 1 mL PBS and transfected with 100 μL of serum-free RPMI 1640 medium containing ASCP NPs (final concentrations: 2 or 4 μg/mL) for 4 h. Then, cells were treated with or without irradiation inspired by an 808 nm laser (0.5 W/cm^2^) for 10 min. After being cultured for 24 h, cells were treated with twice PBS washing and stained with calcein-AM (for live cells: green fluorescence) and PI (for dead cells: red fluorescence). Fluorescence images of live–dead staining of MC38 cells were obtained with a fluorescent microscope (Leica M205 FCA, Wetzlar, Germany) with filters set for FITC and PI. 

### 2.18. Proliferation Inhibition Analysis with or without Irradiation

A CCK-8 assay was employed to carry out the proliferation inhibition analysis. MC38 cells were seeded into 96-well plates at a density of 1 × 10^4^ cells/well and incubated overnight for adherence. Subsequently, cells were treated with 100 μL RPMI 1640 complete medium with different concentrations of ASCP NPs (0, 0.25, 0.5, 1, 2, 4, 8, 16 μg/mL). Cells treated with an equal volume of PBS were used as the control group. After a 4-h treatment with NPs, MC38 cells were irradiated with or without an 808 nm laser (0.5 W/cm^2^) for 10 min. After continuing the culture for another 20 h, the cells were added with 10 μL CCK-8 in each well and then incubated for 2 h at 37 °C. Finally, the cell viability was evaluated with a CCK-8 kit by measuring the absorbance of 450 nm (*A*_450_) and calculated as follows: cell viability (%) = *A*_450_ of test well/*A*_450_ of PBS well without irradiation × 100%.

### 2.19. Gene Expression Assay

After being cultured overnight, MC38 cells seeded in 6-well plates (5 × 10^5^ cells per well) were transfected with PBS, siPD-L1 only, ASCP/siNC NPs, ASCP/siPD-L1 NPs and Lipo3000/siPD-L1 NPs for 4 h following the methods described in the above siRNA transfection. The final transfected concentration of ASCP NPs was 2 μg/mL and the dose of siRNA was 50 pmol. Next, the cells were irradiated with or without the 808 nm laser (0.5 W/cm^2^) for 10 min and cultured for another 20 h. Then, the transcription level of the PD-L1 gene of the 24 h post-transfection MC38 cells was investigated using quantitative real-time polymerase chain reaction (qPCR) according to previous experience [46]. Briefly, the total RNA was extracted from MC38 cells using a TRIzol reagent and quantified using a micro-spectrophotometer (EPOCH-2, Bio-Tek Instruments, Winooski, VT, USA). Then, the total RNA (1 μg) was reversely transcribed to complementary deoxyribonucleic acid (cDNA) by using the QuantiTect Reverse Transcription Kit in accordance with the manufacturer’s instructions. Finally, the mRNA level of the PD-L1 gene was measured by a qPCR instrument (QuantStudio-1, Appliedbio systems, Waltham, MA, USA) by using the Bestar SybrGreen qPCR Master Mix, followed by normalizing to the expression of GAPDH. 

### 2.20. In Vitro Killing Effect of Co-Incubation on MC38 Cells

In order to simulate the T-cell-mediated anti-cancer immune response in vitro, the killing effects of co-incubation of BMDCs and splenocytes on ASCP-based NPs-treated MC38 cells were detected with or without laser irradiation. Briefly, MC38 cells in the logarithmic phase were trypsinized, collected and stained with CFSE (5 μM) at 37 °C for 10 min under gentle shaking. After washing them twice with an equal volume of PBS, the CFSE labeled cells were resuspended in the RPMI 1640 complete medium and seeded in 12-well plates at 1 mL volume with 1 × 10^5^ cells. After being cultured overnight for adherent, the MC38 cells were transfected with PBS, CpG (1 mg/mL), ASCP (1 μg/mL), ASCP (2 μg/mL), ASCP/siPD-L1 (1 μg/mL by ASCP mass) and L1 ASCP/siPD-L1 (2 μg/mL by ASCP mass) for 4 h. Then, the MC38 cells treated with or without 10-min mild photothermal heating inspired by an 808 nm laser (0.5 W/cm^2^) and cultured for another 4 h. Next, BMDCs and splenocytes were added in each well at the number ratio of effector cells to target cells of 50:1. After being co-incubated with BMDCs and splenocytes for 48 h, the killing effects on MC38 cells in different treatment groups were analyzed by flow cytometry with a PI staining buffer by gating in PI^+^CFSE^+^ cells.

### 2.21. In Vitro Immune Activation Effect Induced by Co-Incubation

A similar experimental condition as described above was used to test the in vitro immune activation effect induced by co-incubation. In brief, after being treated with different formulations of NPs with or without 10-min mild photothermal heating, MC38 cells were co-incubated with BMDCs and splenocytes for 48 h. Then, the co-incubated cells in each group were divided into 2 tubes. For the activation effect detection on BMDCs, the co-incubated cells were stained with the antibodies of Ms CD45 PerCP 30-F11, Ms CD11c APC HL3, Ms CD86 PE GL1 and Ms I-A I-E Alexa 488 M5/114.15.2. Finally, the maturity of BMDCs (CD86^+^MHC-II^+^%) gated in CD45^+^CD11c^+^ cells was measured by flow cytometry to assess the in vitro activation effect of ASCP NPs and ASCP/siPD-L1 NPs on BMDCs. For the activation effect detection on splenic lymphocytes, the co-incubated cells were stained with the antibodies of Ms CD45 PerCP 30-F11, Ms CD3e APC 145-2C11, Ms CD4 PE-Cy7 RM4-5, Ms CD8a FITC 53-6.7 and Ms CD49b PE HM Alp2. Finally, the in vitro immune activation effect induced by co-incubation on splenic lymphocyte differentiation was detected by flow cytometry gated in CD45^+^ cells. The CD3^+^CD4^+^ cells and CD3^+^CD8^+^ cells were effector T lymphocytes, and the CD49b^+^ cells were NKs. 

### 2.22. Statistical Analysis Methods

The number of biological samples in experiments was not less than three (n ≥ 3). Statistical analysis and graphs collection were performed using one-way analysis of variance (ANOVA) with Tukey multi-comparisons by GraphPad Prism v.8.0 (GraphPad Software Inc., San Diego, CA, USA). All data were shown as means ± SD. A *p*-value less than 0.05 was considered statistically significant, and * *p* < 0.05, ** *p* < 0.01, *** *p* < 0.001.

## 3. Results and Discussion

### 3.1. Preparation and Characterization of ASCP/siRNA NPs

AuNRs and AuNRs@MS (AS) were prepared by using the seed-mediated growth method, as reported by Zhang et al. [39,43,44]. Through the successive surface modification steps of adsorbing CpG ODNs and wrapping them with PEG-bPEI, the co-delivery vector ASCP/siPD-L1 was successfully obtained (Scheme 1A). The morphology of NPs synthesized at each step of the preparation process was measured under transmission electron microscopy (TEM). The aspect ratio of the prepared AuNRs was about 4:1 with a particle size of about 80 × 20 nm (Figure S1). AS NPs were coated with MS shells with a thickness of about 40 nm around the AuNRs core (Figure S2). The abundant pores on the surface of MS shells enabled them to be used as carriers to adsorb a large number of small molecules, such as nucleic acid fragments [30]. Therefore, CpG ODNs, as short synthetic single-stranded DNA molecules [47], could be adsorbed into the pores on the MS shells of AS NPs. Next, the loading capacity of CpG ODNs by AS NPs was tested under the different mass ratios of 1.5:1, 3:1, 5:1, 10:1 and 20:1. The electrophoretic mobility results showed that the adsorption capacity of CpG ODNs to AS NPs reached the upper threshold at the mass ratio of 5:1 (AS to CpG ODNs), (Figure S3). The peak value of UV-vis absorbance spectrum at 260 nm indicated that the CpG ODNs were successfully loaded onto AS NPs to form ASC NPs (Figure S4). Then, the CpG ODNs’ loading efficiency was determined, including the CpG entrapment efficiency and the CpG loading content of ASC NPs. The CpG entrapment efficiency of ASC NPs was increased with the increase in weight ratio and began to reach the upper limit of 98.61% when the weight ratio was up to 5:1 (Figure S5). The CpG loading content of ASC NPs was at the highest at the weight ratio of 3:1 and the second highest at the weight ratio of 5:1 (Figure S5). Considering the results of electrophoretic mobility and the CpG ODNs’ loading efficiency, ASC NPs were prepared at an AS/CpG ODNs weight ratio of 5:1. Coating and modification with positive potential cationic polymers (such as PEG-bPEI) are common means to convert nanoparticles into siRNA carriers [48,49]. Finally, ASCP NPs were prepared by mixing ASC NPs with PEG-bPEI at the weight ratio of 5:1 and by night stirring and centrifugation. Overall, all the AS-based NPs exhibited a typical oval shape with a particle size of about 120 × 60 nm in TEM (Figure S6). The pores in the outer MS shell of the initially synthesized AS NPs were clearly visible. However, with the sequential loading of CpG ODNs and PEG-bPEI to form ASC and ASCP NPs, the pore clarity on the MS shell gradually decreased, especially after PEG-bPEI was wrapped (Figure S6). The mean diameter of AS NPs, ASC NPs and ASCP NPs obtained by dynamic light scattering (DLS) in ultra-pure water was 75.45 nm, 84.67 nm and 95.03 nm, respectively (Figure S7). The corresponding average zeta potential values of these NPs were +23.54 mV, −20.67 mV and +39.38 mV, respectively (Figure S8). 

**Figure 1:**
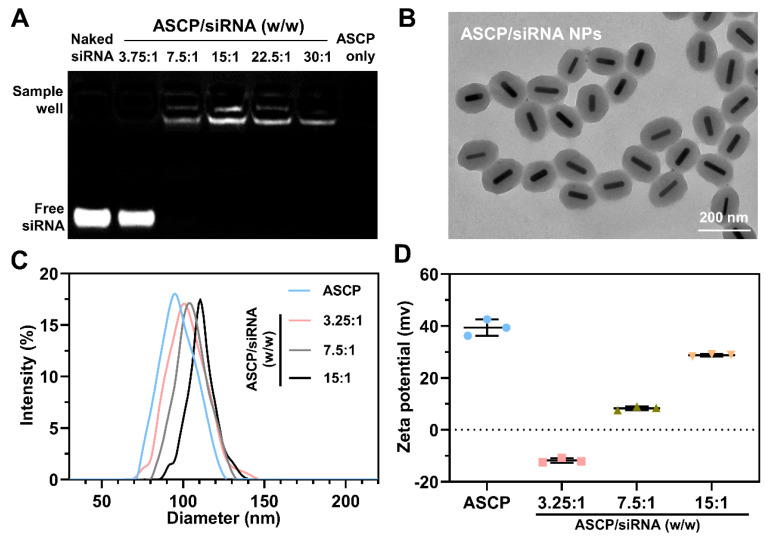
Preparation and characterization of ASCP/siRNA NPs. (**A**) Electrophoretic mobility of ASCP/siRNA NPs at different weight ratios of ASCP to siRNA. The dose of siRNA was fixed at 0.133 µg per well. (**B**) TEM images of the ASCP/siRNA NPs prepared at the weight ratios of 7.5:1 (ASCP to siRNA), scale bar = 200 nm. (**C**) Particle sizes and (**D**) zeta potential values of the ASCP/siRNA NPs prepared at different weight ratios of ASCP to siRNA. All data are presented as the mean ± SD (n = 3).

The positive potential from the PEG-bPEI on the surface of ASCP NPs gave them the ability to adsorb negatively charged siRNA. ASCP/siRNA NPs were formed by incubating siRNA with ASCP NPs through an electrostatic interaction for 60 min. Agarose gel electrophoresis of ASCP/siRNA NPs formed at different weight ratios (ASCP/siRNA = 3.75:1, 7.5:1, 15:1, 22.5:1 and 30:1) was conducted to evaluate the siRNA adsorption capacity of ASCP NPs. As shown in Figure 1A, the amount of unbound siRNA was reduced by increasing the weight ratio of ASCP/siRNA. And the siRNA-binding capacity of ASCP began to reach the maximum at the weight ratio of 7.5:1. The size of ASCP/siRNA NPs formed at such weight ratio was about 120 × 60 nm in elliptical diameter, which was similar to that of ASCP NPs (Figure 1B). The particle sizes and zeta potential values of ASCP/siRNA NPs prepared at different weight ratios in ultra-pure water were measured by the DLS method. The mean particle size of ASCP NPs was 95.03 nm, while that of ASCP/siRNA NPs formed at the weight ratios of 3.75:1, 7.5:1 and 15:1 was 101.93, 104.56 or 109.67 nm, respectively (Figure 1C). The zeta potential values of these corresponding nanoparticles were +39.38 mV, −11.33 mV, +8.32 mV and +28.73 mV (Figure 1D). The results displayed that the particle size and zeta potential values of ASCP/siRNA NPs increased with the weight ratio of ASCP to siRNA, as it is known that the positive zeta potential of nanoparticles is more conducive to the adsorption and uptake of nanoparticles by cells [20,21]. In addition, nanoparticles with a size ranging from 50–150 nm display the enhanced permeability and retention (EPR) effect in tumor tissue [20,21]. Overall, ASCP/siRNA NPs were expected to show high transfection efficiency and enhanced accumulation in tumor sites.

### 3.2. In Vitro Photothermal Effect of ASCP NPs

**Figure 2:**
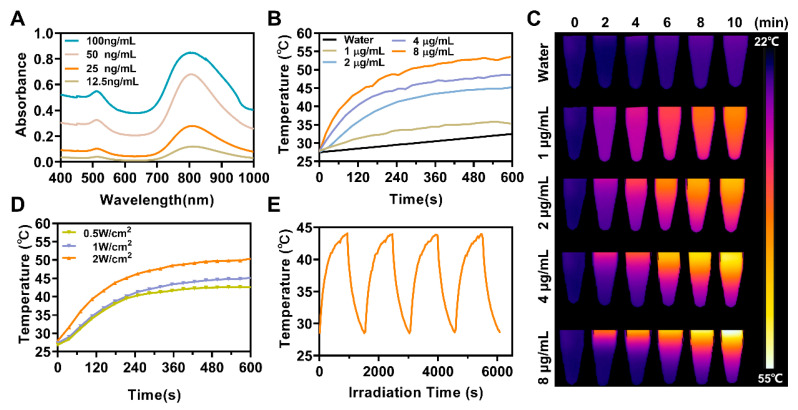
The photothermal response of ASCP NPs. (**A**) The UV-visible spectrums for different concentrations of ASCP NPs. (**B**) The temperature elevation curve and (**C**) the temperature gradience of ASCP NPs in different concentrations under laser irradiation (808 nm, 1 W/cm^2^) for 10 min. (**D**) The temperature elevation curve of ASCP NPs (2 µg/mL) under laser irradiation with different power densities for 10 min. (**E**) Photostability of ASCP NPs (2 µg/mL) evaluated by performing the lasering and cooling cycle several times.

AuNRs@MS usually exhibits excellent photothermal conversion properties in the near infrared-I region (NIR-I, 650–900 nm) [31]. Both the absorption values of AuNRs and AS NPs were concentration-dependent. Compared to AuNRs, the longitudinal plasma formant of AS NPs slightly redshifted from 795 nm to 808 nm, while the transverse plasmon formant was slightly redshifted from 500 nm to 512 nm (Figure S9). This redshift phenomenon was caused by the variation in the refractive index around AuNRs after coating the MS shells, thereby altering the frequency of localized surface plasmon resonance (LSPR). Furthermore, the plasma formant of ASCP NPs was consistent with that of AS NPs, and the absorption value also showed concentration-dependent effects (Figure 2A). The photothermal response of aqueous dispersions of ASCP NPs was then tested by irradiation with an 808 nm laser at a power density of 1 W/cm^2^ for 10 min at room temperature (RT). Both the temperature elevation curve (Figure 2B) and the temperature gradience (Figure 2C) of ASCP NPs showed typical concentration-dependent effects from 1 to 8 µg/mL. The temperature was generally controlled from 42–45 °C during the process of MPTT [40]. The temperature of the 2 µg/mL ACSP solution was maintained at about 43–45 ℃ during the 5–10 min irradiation period (Figure 2B), which met the requirements of MPTT. Next, the results showed that the ASCP temperature (2 µg/mL) was also positively correlated with the irradiation power, as the laser power density increased from 0.5 to 2.0 W/cm^2^ (Figure 2D). More importantly, when the laser power density was 0.5 W/cm^2^, the temperature of the 2 µg/mL ACSP solution could be maintained at about 42–43 °C during the irradiation period of 5–10 min (Figure 2D). In addition, through the repeated measurement of four lasering and cooling cycles, the ACSP solution with the concentration of 2 µg/mL exhibited excellent photothermal stability (Figure 2E). These results indicated that ASCP had superior photothermal properties and could mediate mild photothermal heating under the 808 nm irradiation with the laser power densities of 0.5–1 W/cm^2^ at a concentration of 2 µg/mL. In addition, the milder temperature maintained by the 0.5 W/cm^2^ irradiation was expected to be more beneficial in avoiding damage to normal cells.

### 3.3. In Vitro Biosafety Evaluation of ASCP NPs

Good biosafety is a prerequisite for the clinical application of nanomaterials. The in vitro biosafety of ASCP was evaluated by the hemolysis test and cytotoxicity assay prior to biological experiments. As shown in Figure S10A, the hemolysis rate of ASCP increased with its concentration. When the concentration of ASCP was not more than 2 µg/mL, its hemolysis rate was less than 5%, which meets the relevant international standards. Next, the cytotoxicity of ASCP NPs on the mouse colorectal cancer cell line MC38, mouse breast cancer cell line 4T1 and mouse splenocytes was respectively evaluated by the CCK-8 assay (Figure S10B). The cytotoxicity assay results indicated that the cell viability of MC38 cells and 4T1 cells remained above 80% when the concentration of ASCP was less than 8 µg/mL. However, at high concentrations (≥16 µg/mL), their cell viability decreased rapidly to 70% and 50%, respectively (Figure S10B). Notably, the cell viability of splenocytes remained above 90% when the ASCP concentration was less than 16 µg/mL. Splenocytes are primary cells. The weak cytotoxic effect of ASCP on splenocytes suggested that ASCP might have good biosafety in vivo. Considering the photothermal properties and in vitro biosafety results, the ASCP with a concentration of 2 µg/mL was selected for subsequent tumor cytology experiments.

### 3.4. Mild Photothermal Heating-Enhanced Transfection of ASCP/siRNA NPs 

**Figure 3:**
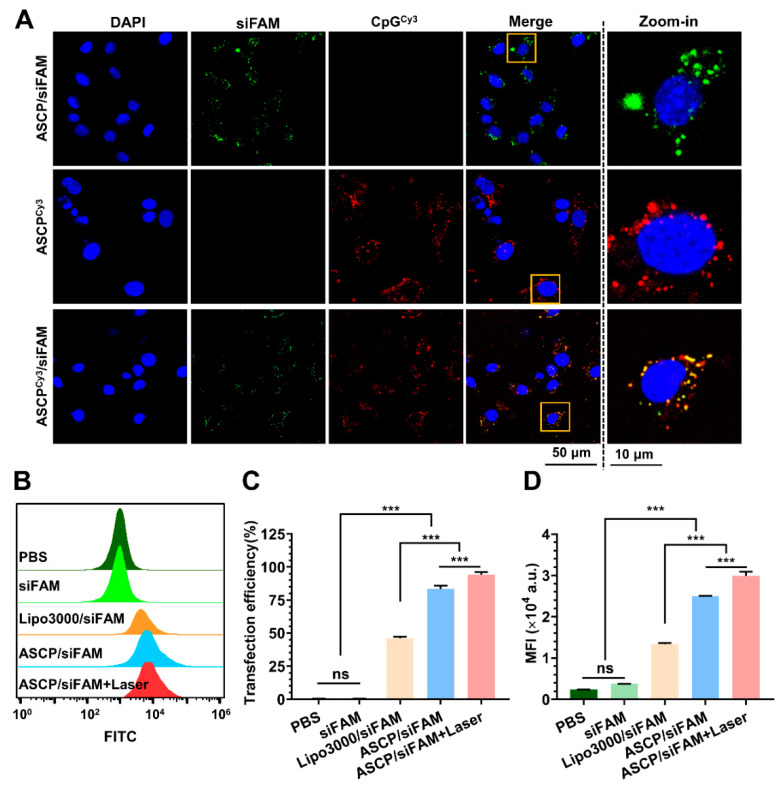
Intracellular uptake of siRNA and CpG ODN co-delivered by ASCP NPs in MC38 cells. (**A**) Fluorescence images of MC38 cells transfected with the NPs of ASCP/siFAM, ASCP^Cy3^ and ASCP^Cy3^/siFAM for 4 h. Blue: cell nuclei were stained by DAPI, green: siRNA labeled with FAM (siFAM), red: CpG ODN labeled with Cy3 loaded in ASCP (ASCP^Cy3^), merge: DAPI + FAM + Cy3, scale bar = 50 μm. (**B**) Flow cytometry detected the transfection efficiency results of ASCP/siFAM NPs prepared at the weight ratio of 7.5:1 in MC38 cells. (**C**) Quantitative analysis of transfection efficiency rates and mean fluorescence intensity (MFI) recorded in panel (**D**). All data are expressed as the mean ± SD (n = 3. Statistics were done using one-way ANOVA with Tukey multi-comparisons. *** *p* < 0.001 and ns, no significant difference).

High transfection efficiency is one of the key characteristics of siRNA delivery systems [20,21,22]. AuNRs@MS-based delivery systems are often used to deliver drugs or genes [43]. The cell uptake of the NPs of ASCP/siFAM, ASCP^Cy3^ and ASCP^Cy3^/siFAM on MC38 cells was observed by the CLSM (Figure 3A). Both CpG^Cy3^ and siFAM could be successfully and efficiently delivered into MC38 cells by ASCP at 4 h after the transfection. The high coincidence of CpG^Cy3^ red fluorescence and siFAM green fluorescence in the ASCP^Cy3^/siFAM group proved that ASCP NPs could be used as co-delivery vectors to efficiently transfect CpG ODNs and siRNA into MC38 cells (Figure 3A). Mild photothermal heating could increase the cell membrane permeability to improve cellular uptake and gene transfection efficiency [40,41]. In addition, the morphology of ASCP NPs before and after irradiation did not change significantly in TEM images, indicating that mild photothermal heating had no significant effect on its stability (Figure S11). Next, the transfection efficiency of ASCP as a co-vector of CpG ODNs and siRNA on MC38 cells was quantified by flow cytometry with or without laser irradiation (Figure 3B). The results showed that the transfection efficiency and MFI of siRNA-FAM delivered by ASCP were 83.29% and 2.496 × 10^4^, respectively, significantly higher than 45.91% and 1.334 × 10^4^ delivered by Lipo3000. Furthermore, the transfection efficiency and the MFI was substantially enhanced to 94.29% and 2.989 × 10^4^ by the mild photothermal heating provided by laser irradiation (808 nm, 0.5 W/cm^2^, 10 min) (Figure 3C,D). These results demonstrated that ASCP could effectively co-transfect CpG ODNs and siRNA into MC38 cells and mediate mild photothermal heating to enhance the transfection effect further.

### 3.5. Mild Photothermal Heating-Enhanced Endo-Lysosomal Escape of ASCP/siRNA NPs

**Figure 4:**
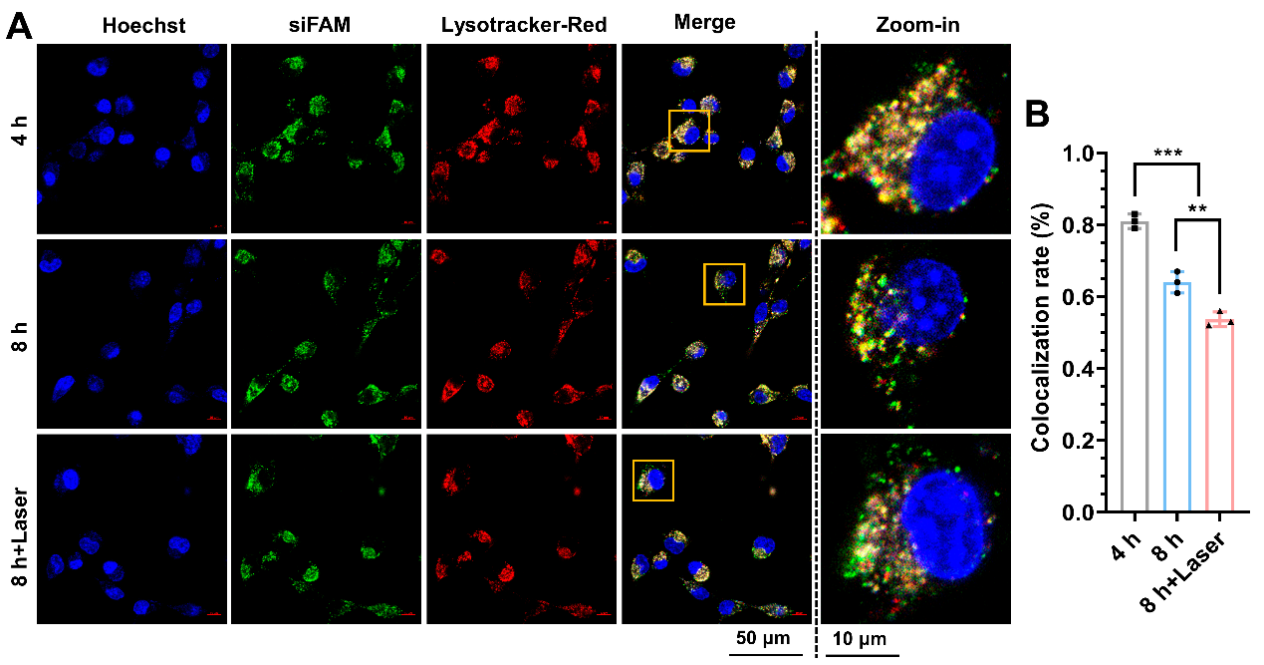
Endo-lysosome escaping and colocalization analysis of ASCP/siRNA NPs in MC38 cells. (**A**) Subcellular localization of ASCP/siRNA NPs in MC38 cells at 4 h or 8 h post-transfection with or without laser radiation. Blue: cell nuclei were stained by Hoechst 33342, red: endosomes or lysosomes stained by lysotracker red, green: siRNA labeled with FAM (siFAM), merge: Hoechst + lysotracker red + FAM, scale bar = 50/10 μm. (**B**) Colocalization ratios of ASCP/siRNA-FAM formulations and lysotracker red-stained endo-lysosomes at different transfection time points with or without laser radiation. All data are expressed as the mean ± SD (n = 3. Statistics were done using one-way ANOVA with Tukey multi-comparisons. ** *p* < 0.01 and *** *p* < 0.001).

Numerous reports provide evidence that only 1–3.5% of siRNA endocytosed by cells could successfully escape from the endo-lysosome and trigger RNAi [50]. Most of the remaining siRNA that did not escape were rapidly degraded in the acidic environment of endo-lysosome [51,52], which limited the initiation of RNAi to effectively silence target genes, resulting in unsatisfactory therapeutic effects [53]. Therefore, effective siRNA endo-lysosomal escape is important for improving the gene silencing efficiency and therapeutic effect [20]. To investigate the siRNA endo-lysosome escape efficiency assisted by ASCP, the subcellular localization of ASCP/siFAM NPs in MC38 cells with or without irradiation was observed under a CLSM (Figure 4A). The statistics in Figure 4B showed that the mean colocalization coefficient of siRNA and endo-lysosome of ASCP/siFAM NPs was 0.810 at 4 h post-transfection. In contrast, the colocalization coefficient decreased to 0.640 at 8 h after transfection. Notably, an additional 10 min-mild photothermal heating condition provided by the 808 nm laser (0.5 W/cm^2^) further reduced the colocalization coefficient to 0.537 (Figure 4B). The endo-lysosomal escape efficiency was negatively correlated with the colocalization coefficient. These results have proven that ASCP could not only effectively assist siRNA in achieving endo-lysosomal escape, but also mediate mild photothermal heating to enhance endo-lysosomal escape further.

### 3.6. In Vitro Immune Activation Effect of ASCP/siRNA NPs

**Figure 5:**
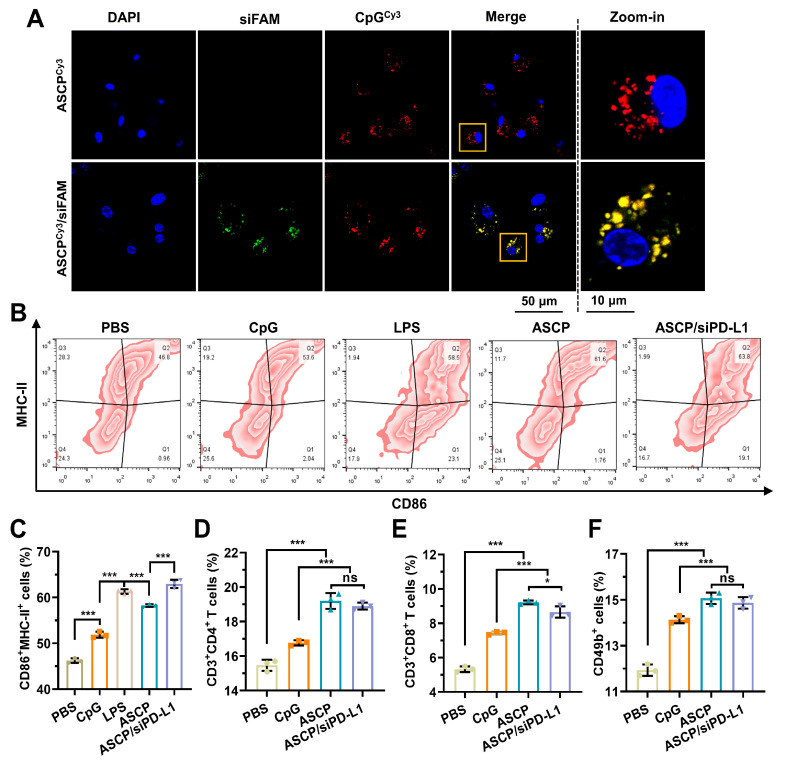
Cellular uptake and in vitro activation effect of ASCP NPs and ASCP/siPD-L1 NPs on BMDCs. (**A**) Fluorescence images of BMDCs transfected with ASCP^Cy3^ NPs or ASCP^Cy3^/siFAM NPs for 4 h. Blue: cell nuclei were stained by DAPI, green: siRNA labeled with FAM (siFAM), red: CpG ODN labeled with Cy3 loaded in ASCP (ASCP^Cy3^), merge: DAPI + FAM + Cy3, scale bar = 50 μm. The representative flow cytometric detection (**B**) and statistical data (**C**) show BMDCs differentiation induced by different formulations of NPs. CD86^+^MHC-II^+^ cells gated in CD45^+^CD11c^+^ cells were mature DCs. The flow cytometric statistical data (**D**–**F**) show splenic lymphocytes’ differentiation induced by different formulations of NPs. CD3 was the marker of T lymphocytes, CD4 and CD8 were the markers of effector T lymphocytes, and CD49b was the marker of NKs. All data are expressed as the mean ± SD (n = 3. Statistics were done using one-way ANOVA with Tukey multi-comparisons. * *p* < 0.05, *** *p* < 0.001 and ns, no significant difference).

As professional antigen presenting cells (APCs), mature DCs bridge innate immunity and adaptive immunity by presenting antigens to lymphocytes [54]. The activation of DC maturation is necessary for T cell-mediated anti-cancer immunity [55]. CpG ODNs can be recognized and bound by TLR9 expressed by DCs, which promotes the maturation and antigen presentation ability of DCs and initiates a T cell-mediated anti-cancer immune response [24]. ASCP-based NPs were expected to promote DC maturation and initiate the T cell-mediated anti-cancer immune response by releasing CpG ODN. To test the activation effect of ASCP-based NPs on DCs, mouse BMCs were isolated and co-induced to prepare BMDCs using IL-4 and GM-CSF according to the method reported by Tang et al. [45]. After induction, the changes in the cell morphology (Figure S12) and the significant increase in CD11c^+^ expression detected by flow cytometry (Figure S13) indicated that BMDCs were successfully prepared. Then, confocal fluorescence images obtained after BMDCs were co-incubated with ASCP^Cy3^/siFAM NPs for 4 h, which indicated that these NPs could be successfully taken up by the DCs (Figure 5A). Next, the different formulations of NPs were incubated with BMDCs for 24 h with LPS as the positive control. Furthermore, the effects of these NPs on activating BMDCs maturation were evaluated by flow cytometry analysis with CD86 and MHC-Ⅱ as the surface markers for mature DCs (Figure 5B). The maturity of BMDCs (CD86^+^MHC-II^+^%) in the negative control PBS group was 46.23%, while that in the groups of CpG ODN alone and LPS were 51.87% and 58.23%, respectively. However, after the treatments with ASCP NPs and ASCP/siPD-L1 NPs, the DC maturity could be significantly increased to 61.30% and 62.97%, respectively (Figure 5C). These results indicated that ASCP NPs and ASCP/siPD-L1 NPs could not only be effectively take up by DCs, but also had stronger abilities to stimulate DC maturation compared to CpG ODN only or positive stimulant LPS.

Mature DCs can effectively process and present TTAs to T lymphocytes, promote the rapid proliferation and infiltration of T lymphocytes, and initiate the T cell-mediated anti-cancer immune response [55,56]. The spleen, as the largest immune organ and one of the main settlements of lymphocytes, contains a large number of lymphocytes and a small number of DCs [57]. After co-incubation with splenocytes for 48 h, the activation effect of different formulations of NPs on lymphocyte differentiation was detected by flow cytometry (Figure S14). Statistics showed that the average rates of CD3^+^CDD4^+^ T cells in splenocytes treated with PBS and CpG ODN were 15.47% and 16.77%, while those of splenocytes treated with ASCP NPs and ASCP/siPD-L1 NPs were significantly increased to 19.2% and 18.99%, respectively (Figure 5D). Furthermore, the average rates of CD3^+^CD8^+^ T cells and CD49b^+^ cells in the corresponding groups were 5.33%, 7.43%, 9.12%, 8.82% and 11.93%, 14.13%, 15.07% and 14.87%, respectively (Figure 5E,F). These data proved that ASCP-based NPs could significantly activate lymphocyte differentiation into CD4^+^ effector T lymphocytes, CD8^+^ effector T lymphocytes and NKs, which were stronger than that of CpG ODN alone. Taken together, ASCP-based NPs could be effectively uptaken by DCs to activate the maturation of DCs. The resulting mature DCs presented antigens to the T lymphocytes to activate the differentiation and amplify the effector T lymphocytes and NKs, which was expected to initiate the T cell-mediated anti-cancer immune response.

### 3.7. In Vitro Mild Photothermal Heating-Enhanced Gene/Immunotherapy for Colorectal Cancer

**Figure 6:**
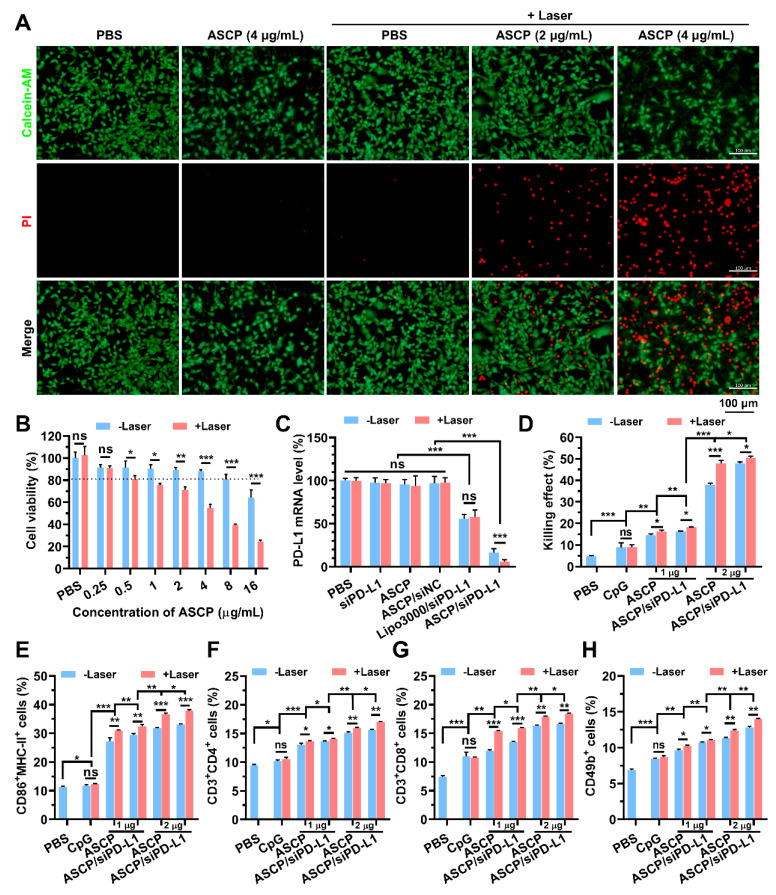
ASCP/siPD-L1 NPs-mediated mild photothermal enhancer gene/immunotherapy for colorectal cancer in vitro. (**A**) Fluorescence images of live–dead staining of MC38 cells treated with different concentrations of ASCP NPs with or without laser irradiation. Green: live cells stained with calcein-AM, red: dead cells stained with PI, merge: calcein-AM + PI, scale bar = 100 μm. (**B**) Cell viability of MC38 cells treated with different concentrations of ASCP NPs with or without laser irradiation. (**C**) The PD-L1 mRNA expression level in ASCP NPs- or ASCP/siPD-L1 NPs-treated MC38 cells with or without laser radiation. (**D**) The killing effect on MC38 cells in different treatment groups after 48 h of co-incubation with BMDCs and splenocytes with or without laser irradiation. The flow cytometric statistical data to show BMDCs maturity (**E**) and splenic lymphocytes’ differentiation (**F**–**H**) in different treatment groups. CD86^+^MHC-II^+^ cells gated in CD11c^+^ cells were mature DCs. CD3 was the marker of T lymphocytes, CD4 and CD8 were the markers of effector T lymphocytes and CD49b was the marker of NKs. All data are expressed as the mean ± SD (n = 3. Statistics were done using one-way ANOVA with Tukey multi-comparisons. * *p* < 0.05, ** *p* < 0.01, *** *p* < 0.001 and ns, no significance).

Due to the excellent photothermal conversion performance of ASCP NPs, the killing effect of ASCP-based PTT on MC38 cells was evaluated (Figure 6A,B). As shown in Figure 6A, ASCP NPs of 4 μg/mL displayed a stronger tumor-killing ability than that of the 2 μg/mL under 10-min irradiation inspired by the 808 nm laser (0.5 W/cm^2^). Thus, the tumor-killing effect of ASCP-based PTT showed a concentration-dependent manner. Previous data showed that the 2 µg/mL ASCP mediated mild photothermal heating (42–43 ℃) under 0.5 W/cm^2^ 808 nm laser irradiation (Figure 2D). Such mild photothermal heating was expected to be more beneficial for avoiding damage to normal cells, promoting cell uptake and facilitating the survival and release of TAAs [40,41,42]. Therefore, the mild photothermal heating-enhanced anti-tumor effect of ASCP NPs was evaluated in vitro at the concentration of 2 μg/mL.

Encouraged by the enhanced transfection efficiency (Figure 3B,D) and endo-lysosomal escape abilities (Figure 4A,B) of ASCP/siRNA NPs under mild photothermal heating, the NIR light-enhanced PD-L1 gene silencing mediated by ASCP/siPD-L1 was further investigated by qPCR on MC38 cells. After a 48 h transfection with or without the 10-min mild photothermal heating inspired by the 808 nm laser (0.5 W/cm^2^), the treated cells showed no significant difference in the PD-L1 mRNA level between the groups of PBS (negative control), siPD-L1, ASCP and ASCP/siNC (Figure 6C). However, the PD-L1 mRNA levels of cells treated with Lipo3000/siPD-L1 and ASCP/siPD-L1 were significantly reduced to 55.77% and 16.47%, respectively. More importantly, the 10-min laser mild photothermal heating further promoted the reduction in the PD-L1 mRNA level in the ASCP/siPD-L1 group to 5.83%, but did not affect that in the Lipo3000/siPD-L1 group (Figure 6C). These data demonstrated that the mild photothermal heating mediated by ASCP/siPD-L1 could not only kill tumor cells, but also enhance the gene silencing efficiency of PD-L1.

PD-L1 blocking can relieve T cell exhaustion and inhibit the immune escape abilities of tumor cells, thus achieving an efficient anti-tumor immunotherapy effect [7,8,9]. PD-L1 silencing can effectively promote the massive differentiation and proliferation of effector T lymphocytes to inhibit the tumor growth and invasion of CRC via similar immunotherapeutic mechanisms [14,15,18,19]. In addition, CpG ODN transfected and released by ASCP-based NPs could effectively promote DC maturation and antigen presentation to initiate a T cell-mediated anti-cancer immune response (Figure 5 and Figure S14). In addition, survival TTAs released during the tumor-killing process of PTT further promote the DC maturation and subsequent T cell-mediated anti-cancer immune response [40,42]. Taken together, ASCP/siPD-L1 was expected to drive a strong anti-cancer effect by mediating the mild photothermal heating-enhanced gene/immunotherapy. In order to simulate the T cell-mediated anti-cancer immune response in vitro, the killing effects of co-incubation of BMDCs and splenocytes on ASCP-based NPs-treated MC38 cells were detected with or without laser irradiation. Briefly, MC38 cells pre-stained with CFSE were transfected with different formulations of NPs with or without 10-min of mild photothermal heating inspired by the 808 nm laser (0.5 W/cm^2^). After 48 h of co-incubation with BMDCs and splenocytes, the killing effects on CFSE-pre-stained MC38 cells in different treatment groups were analyzed by flow cytometry with a PI staining buffer (Figure S15). As the statistical data showed in Figure 6D, the killing effects (CFSE^+^PI^+^%) in groups of PBS, CpG and CpG + Laser were 4.75%, 8.90% and 9.09%, respectively, while the killing effect in groups of ASCP (1 μg/mL), ASCP/siPD-L1 (1 μg/mL), ASCP (2 μg/mL) and ASCP/siPD-L1 (2 μg/mL) were significantly increased to 14.68%, 16.2%, 37.97% and 47.88%, respectively. More importantly, the 10-min mild photothermal heating further promoted the killing effect in the corresponding ASCP-based groups to 16.18%, 18.18%, 47.98% and 50.32%, respectively (Figure 6D).

The BMDCs’ maturity and splenic lymphocytes differentiation in the indicated treatment groups were then analyzed by flow cytometry (Figure S16–S19). The statistics in Figure 6E showed that the corresponding maturities (CD86^+^MHC-II^+^%) of BMDCs in the groups of PBS, CpG, CpG + Laser, ASCP (1 μg/mL), ASCP (1 μg/mL) + Laser, ASCP (2 μg/mL), ASCP (2 μg/mL) + Laser, ASCP/siPD-L1 (1 μg/mL), ASCP/siPD-L1 (1 μg/mL) + Laser, ASCP/siPD-L1 (2 μg/mL) and ASCP/siPD-L1 (2 μg/mL) + Laser were 11.30%, 11.77%, 12.24%, 27.23%, 30.92%, 31.82%, 36.57%, 29.43%, 32.50%, 33.07% and 37.83%, respectively. Correspondingly, the rates of the CD3^+^CD4^+^ T cells in the above groups were 9.45%, 10.20%, 10.53%, 13.03%, 13.64%, 15.06%, 15.93%, 13.60%, 14.03%, 15.57% and 17.00%, (Figure 6F), and the rates of the CD3^+^CD8^+^ T cells in corresponding groups were 7.43%, 10.97%, 10.80%, 11.97%, 15.4%, 16.33%, 17.87%, 13.50%, 15.89%, 16.61% and 18.40%, respectively (Figure 6G). Finally, the corresponding NKs rate (CD49b^+^%) in each group was 6.85%, 8.47%, 8.68%, 9.63%, 10.18%, 11.33%, 12.38%,10.74%, 11.05%, 12.84% and 14.01%, respectively (Figure 6H). From a comprehensive analysis of these data, it can be observed that the DCs maturation and immune activation promoted by ASCP was better than CpG ODN alone, while that promoted by ASCP/siPD-L1 was better than ASCP alone, in a concentration-dependent manner. More notably, the additional 10-min mild photothermal heating could further enhance the role of ASCP-based NPs in promoting DCs maturation and immune activation (Figure 6E–H). Combined with the results of PD-L1 gene silencing and the killing effect on MC38 cells (Figure 6C,D), it can be concluded that the ASCP-mediated mild photothermal heating could not only directly kill MC38 cells and release TTAs from lysates, but also effectively enhance the gene/immunotherapy effect by delivering siPD-L1 and CpG ODN. Eventually, as expected, ASCP/siPD-L1 NPs could effectively kill MC38 cells and have a strong inhibitory effect on CRC under mild photothermal heating.

## 4. Conclusions

In summary, the novel CpG ODN/siRNA co-delivery vectors of ASCP were successfully constructed to mediate mild photothermal heating-enhanced gene/immunotherapy for CRC treatment. By loading CpG ODNs on the AuNRs@MS surface and coating PEG-bPEI and siRNA successively, the multifunctional therapeutic nanocomposites ASCP/siPD-L1 were successfully prepared. ASCP/siPD-L1 NPs exhibited excellent biosafety and promoted the maturity and antigen presentation ability of DCs by delivering CpG ODNs. Next, ASCP/siPD-L1 NPs could not only mediate MPTT to kill tumor cells directly, but also facilitated the release of TTAs from tumor cell lysates to promote the DC maturation and antigen presentation further. Furthermore, mild photothermal heating significantly enhanced the transfection efficiency and endo-lysosomal escape ability of ASCP/siPD-L1 NPs, resulting in an enhanced PD-L1 gene silencing effect. The enhanced DC maturity and PD-L1 gene silencing effectively promoted the proliferation and infiltration of effector T lymphocytes and NKs in TME, thus reversing T cell depletion and inhibiting tumor immune escape. Finally, the combination of MPTT and mild photothermal heating-enhanced gene/immunotherapy could effectively kill MC38 cells, leading to the strong inhibition of CRC. Overall, this work presented high-performance mild photothermal/gene/immune synergies to fight CRC, which provided a pathway for the clinical application of the MPTT-augmented PD-L1 silencing-based gene/immunotherapy.

## Data Availability

All data generated or analyzed during this study are included in this published article and its Supplementary Materials.

## Supplementary Materials

The following supporting information can be downloaded at: https://www.mdpi.com/article/10.3390/pharmaceutics15030854/s1, Figure S1: title TEM images of the gold nanorods (AuNRs), scale bar = 500/200 nm; Figure S2: TEM images of the AS NPs, scale bar = 500/200 nm; Figure S3: Electrophoretic mobility of ASC NPs at different weight ratios of AS to CpG ODN; Figure S4: UV-vis absorbance spectra of CpG ODN only (CpG), AS NPs and ASC NPs; Figure S5: The CpG loading content and CpG loading entrapment efficiency in ASC NPs at different weight ratios of AS to CpG ODN; Figure S6: TEM images of the AS NPs, ASC NPs and ASCP NPs, scale bar = 200 nm; Figure S7: Particle sizes of AS NPs, ASC NPs and ASCP NPs; Figure S8: The zeta potential values of AS NPs, ASC NPs and ASCP NPs; Figure S9: UV-vis absorbance spectra of AuNRs and AS NPs; Figure S10: (A) Hemolysis assay of ASCP with different concentrations. (B) Cytotoxicity analysis of splenocyte, MC38 cells and 4T1 cells treated with different concentrations of ASCP, respectively; Figure S11: TEM images of ASCP NPs before and after laser irradiation, scale bar = 200 nm; Figure S12: Differentiation of bone marrow cells (BMCs) into bone marrow-derived dendritic cells (BMDCs) induced by GM-CSF and IL-4 in vitro; Figure S13: (A) Representative flow cytometric detection and (B) the statistical data to show the differentiation of bone marrow cells (BMCs) into bone marrow-derived dendritic cells (BMDCs) induced by GM-CSF and IL-4 in vitro; Figure S14: Representative flow cytometric detection (A–C) to show splenic lymphocytes differentiation induced by different formulations of NPs; Figure S15: MC38 cells treated with different formulations of NPs with or without laser radiation were co-incubated with BMDCs and splenocytes for 48 h, then the killing effect on MC38 cells was analyzed with flow cytometry by gating in CFSE^+^PI^+^; Figure S16: MC38 cells treated with different formulations of NPs with or without laser radiation were co-incubated with BMDCs and splenocytes for 48 h, then the maturity of BMDCs was analyzed by flow cytometry; Figure S17: MC38 cells treated with different formulations of NPs with or without laser radiation were co-incubated with BMDCs and splenocytes for 48 h, then the differentiation of splenic lymphocytes was analyzed by flow cytometry; Figure S18: MC38 cells treated with different formulations of NPs with or without laser radiation were co-incubated with BMDCs and splenocytes for 48 h, then the differentiation of splenic lymphocytes was analyzed by flow cytometry; Figure S19: MC38 cells treated with different formulations of NPs with or without laser radiation were co-incubated with BMDCs and splenocytes for 48 h, then the differentiation of splenic lymphocytes was analyzed by flow cytometry.
