# Supplementary material for: Multifunctional Mesoporous Silica-Coated Gold Nanorods Mediate Mild Photothermal Heating-Enhanced Gene/Immunotherapy for Colorectal Cancer
Source: Pharmaceutics. 2023 Mar 6;15(3):854. doi: 10.3390/pharmaceutics15030854 (PMC10057058; doi:10.3390/pharmaceutics15030854)
Supplement: Supplementary file 1 [file pharmaceutics-15-00854-s001.zip › pharmaceutics-2238929-supplementary.pdf]

SUPPLEMENTARY MATERIALS

Supplementary Figures

Multifunctional Mesoporous Silica-Coated Gold Nanorods Mediate Mild Photothermal Heating-Enhanced Gene/Immunotherapy for Colorectal Cancer

Meirong Li [1,2,†], Jingyu Yang [1,†], Xinhuang Yao [2], Xiang Li [1], Zhourui Xu [1], Shiqi Tang [1], Bangxu Sun [2], Suxia Lin [3], Chengbin Yang [1,*] and Jia Liu [2,*]

[1] School of Biomedical Engineering, Shenzhen University Medical School, Shenzhen University, Shenzhen 518060, China; mediali19851119@163.com (M.L.); weimor_yang@163.com (J.Y.); 2070246087@email.szu.cn (X.L.); xuzhouray@szu.edu.cn (Z.X.); tsq2899498468@163.com (S.T.)

[2] Central Laboratory of The Second Affiliated Hospital, School of Medicine, The Chinese University of Hong Kong, Shenzhen & Longgang District People's Hospital of Shenzhen, Shenzhen 518172, China; yxhh25@126.com (X.Y.); sbx15645539691@163.com (B.S.)

[3] Center of Assisted Reproduction and Embryology, The University of Hong Kong-Shenzhen Hospital, Shenzhen 518048, China; linsx@hku-szh.org

* Correspondence: cbyang@szu.edu.cn (C.Y.); liujia870702@126.com (J.L.)

† These authors contributed equally to this work.

Supplementary Figures

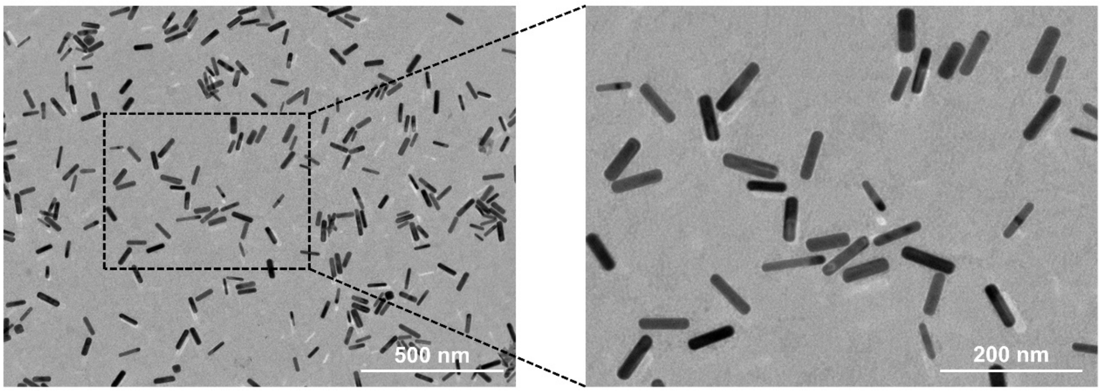

Figure S1. TEM images of the gold nanorods (AuNRs), scale bar = 500/200 nm.

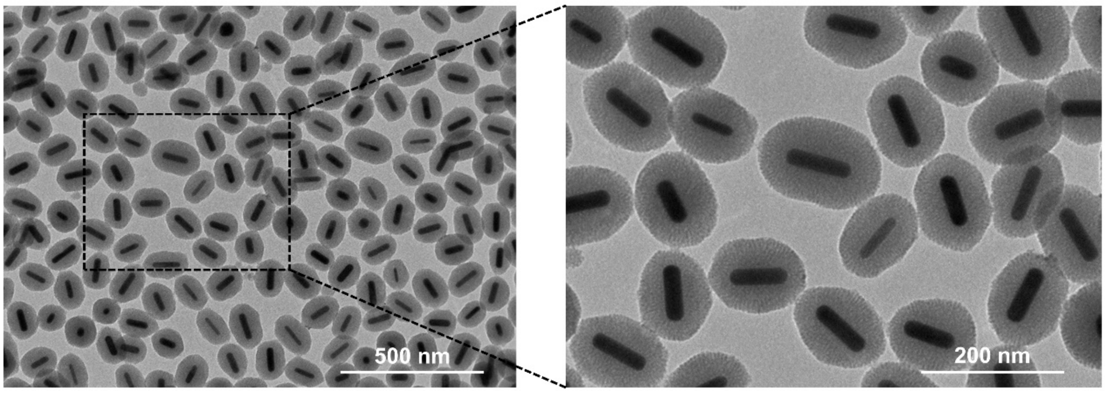

Figure S2. TEM images of the AS NPs, scale bar = 500/200 nm.

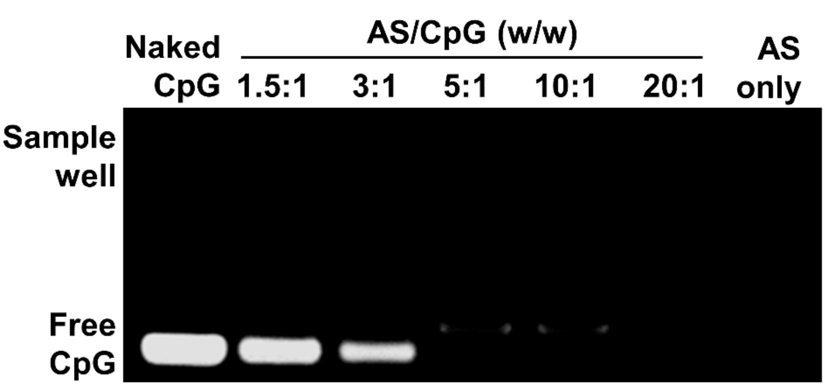

Figure S3. Electrophoretic mobility of ASC NPs at different weight ratios of AS to CpG ODN. The dose of CpG ODN was fixed at 0.2 μg per well.

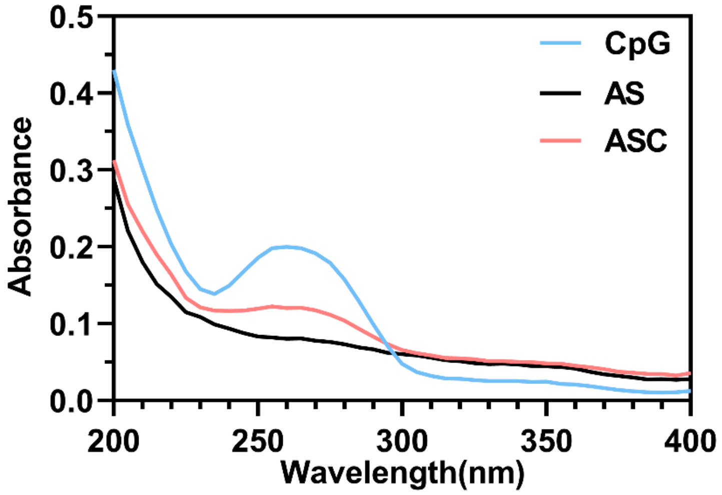

Figure S4. UV-vis absorbance spectra of CpG ODN only (CpG), AS NPs and ASC NPs.

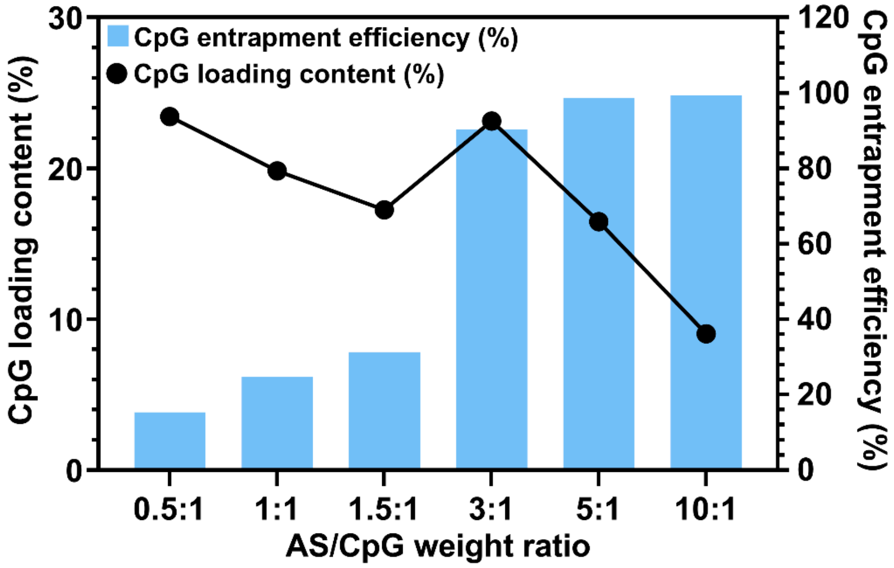

Figure S5. The CpG loading content and CpG loading entrapment efficiency in ASC NPs at different weight ratios of AS to CpG ODN.

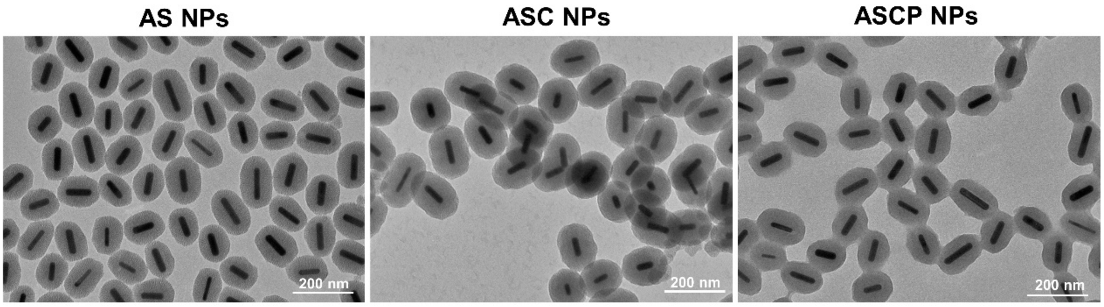

Figure S6. TEM images of the AS NPs, ASC NPs and ASCP NPs, scale bar = 200 nm.

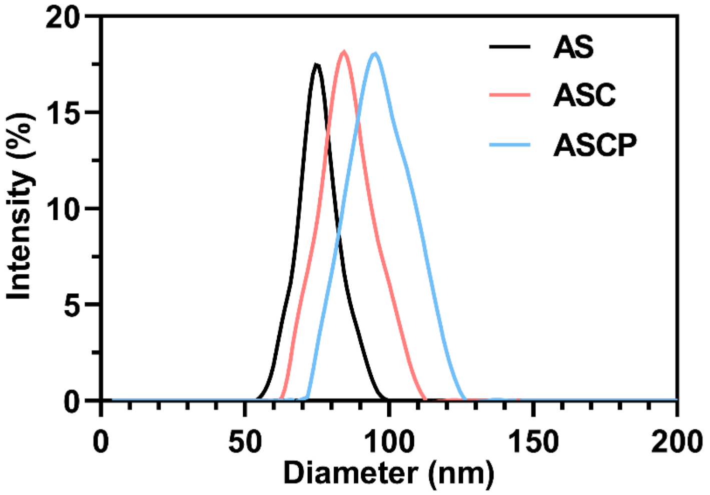

Figure S7. Particle sizes of AS NPs, ASC NPs and ASCP NPs. All data were presented as the mean ± SD (n = 3).

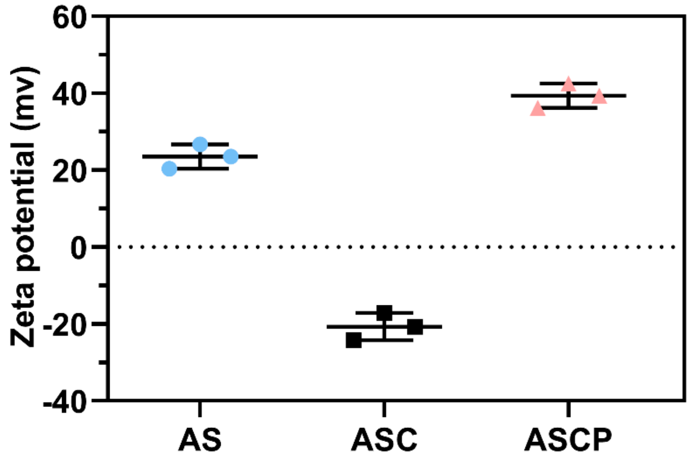

Figure S8. The zeta potential values of AS NPs, ASC NPs and ASCP NPs. All data were presented as the mean ± SD (n = 3).

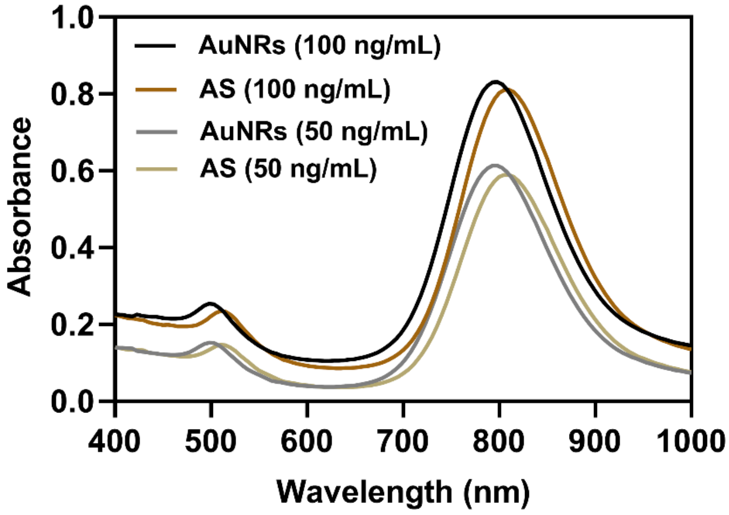

Figure S9. UV-vis absorbance spectra of AuNRs and AS NPs.

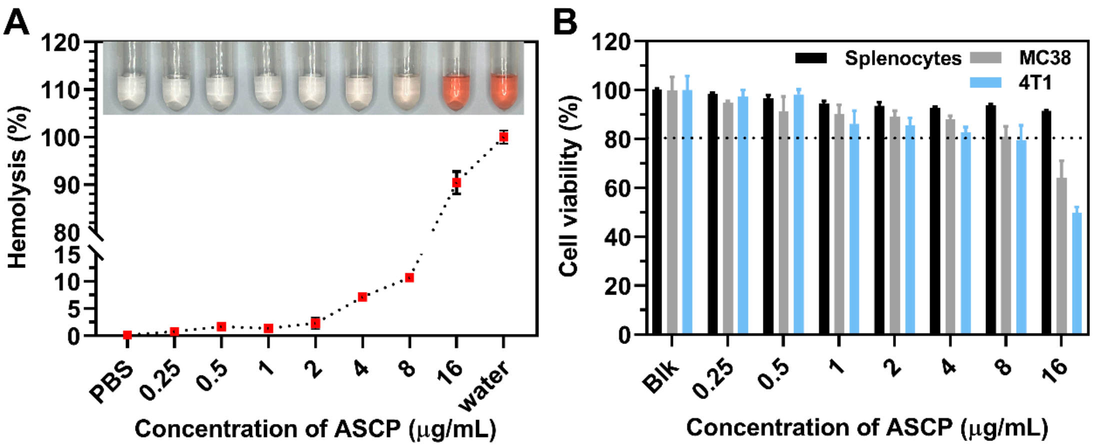

Figure S10. (A) Hemolysis assay of ASCP with different concentrations. (B) Cytotoxicity analysis of splenocyte, MC38 cells and 4T1 cells treated with different concentrations of ASCP, respectively. All data were expressed as the mean ± SD, n = 3.

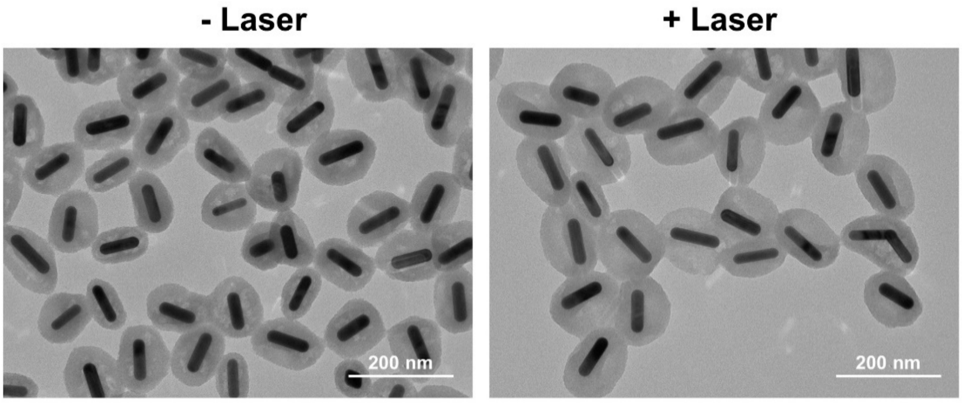

Figure S11. TEM images of ASCP NPs before and after laser irradiation, scale bar = 200 nm.

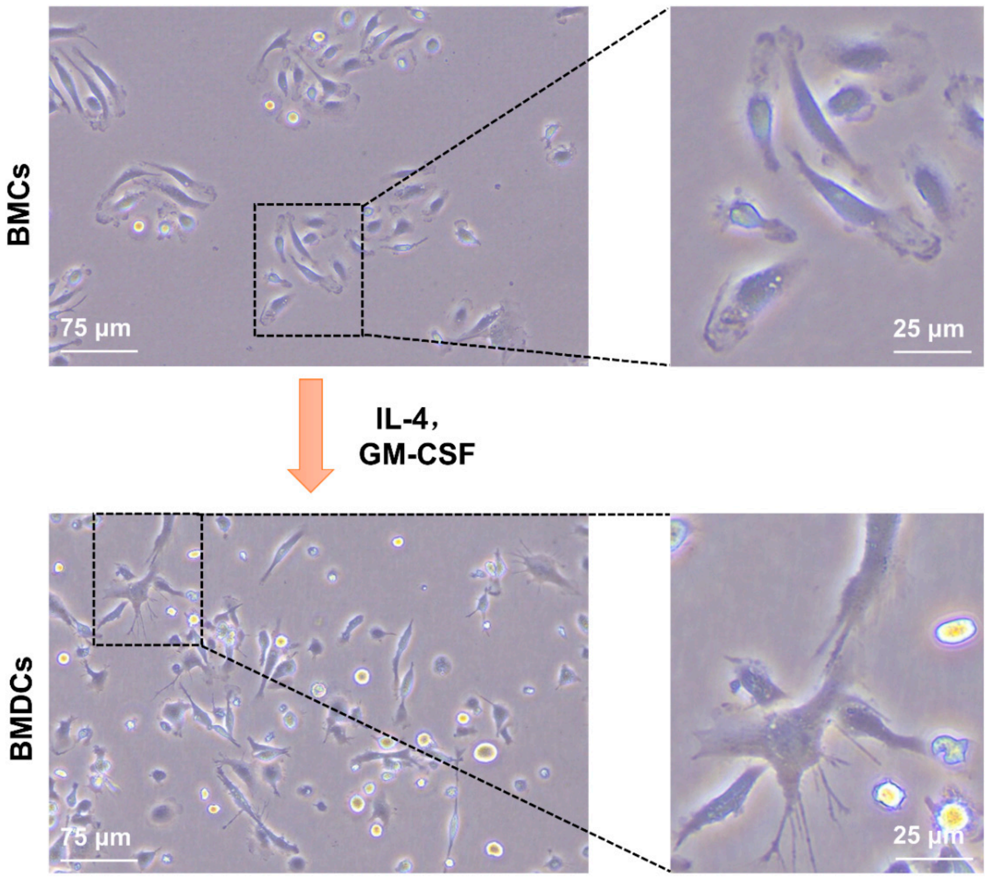

Figure S12. Differentiation of bone marrow cells (BMCs) into bone marrow-derived dendritic cells (BMDCs) induced by GM-CSF and IL-4 in vitro.

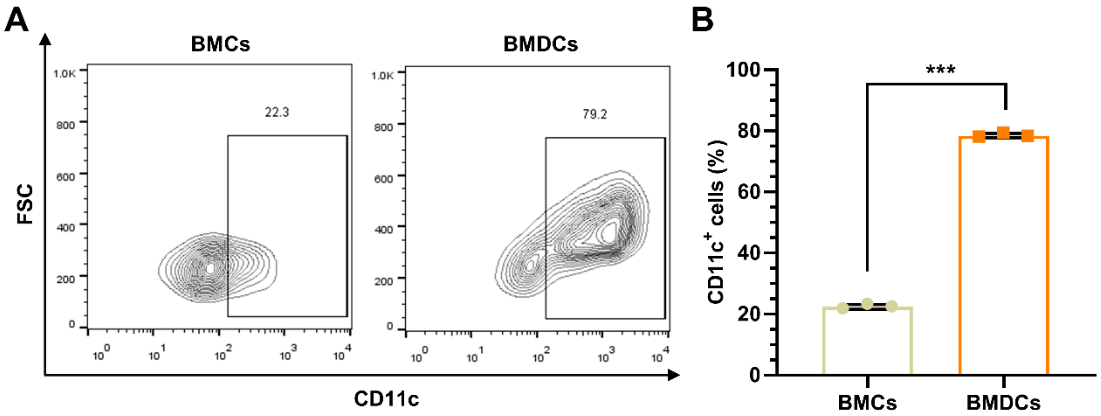

Figure S13. (A) Representative flow cytometric detection and (B) the statistical data to show the differentiation of bone marrow cells (BMCs) into bone marrow-derived dendritic cells (BMDCs) induced by GM-CSF and IL-4 in vitro. CD11c was the marker of DCs. All data were expressed as the mean ± SD (n = 3. Statistics were done using one-way ANOVA with Tukey multi-comparisons. ***p < 0.001).

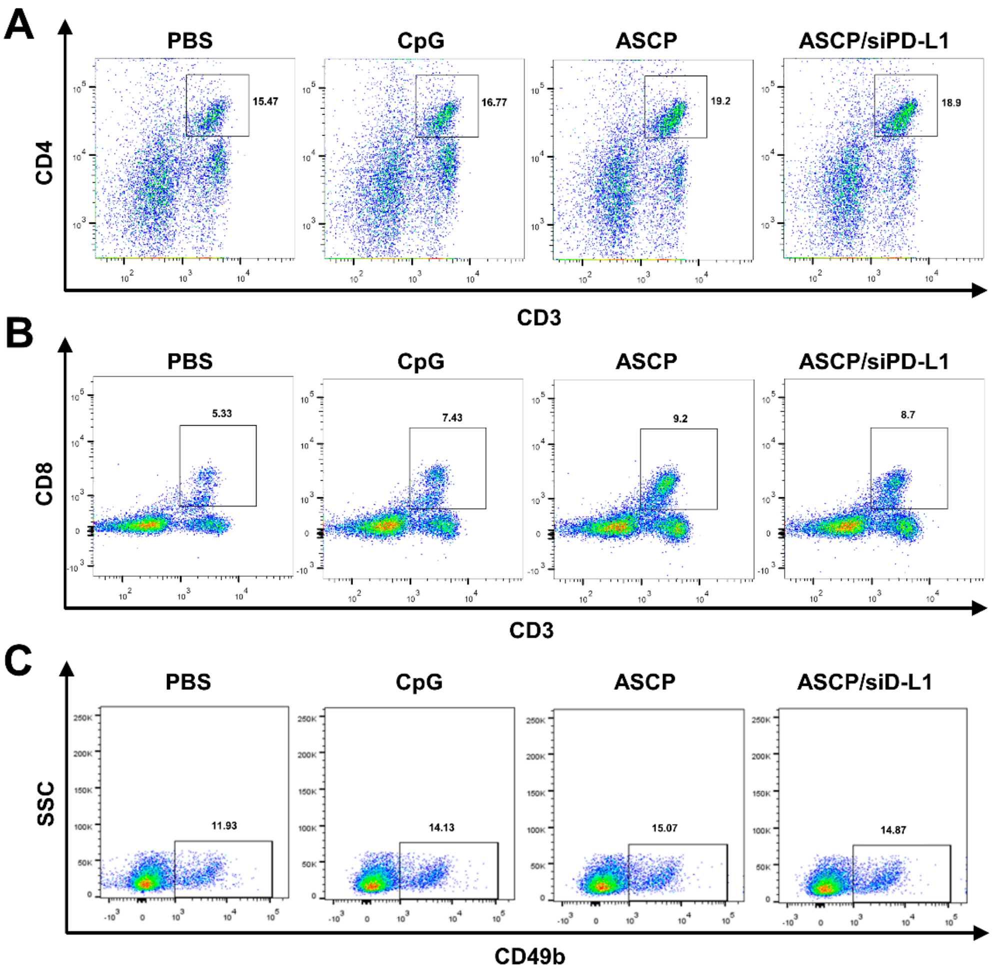

Figure S14. Representative flow cytometric detection (A, B and C) to show splenic lymphocytes differentiation induced by different formulations of NPs. CD3 was the marker of T lymphocytes, CD4 and CD8 were the markers of effector T lymphocytes, and CD49b was the marker of natural killer cells (NKs).

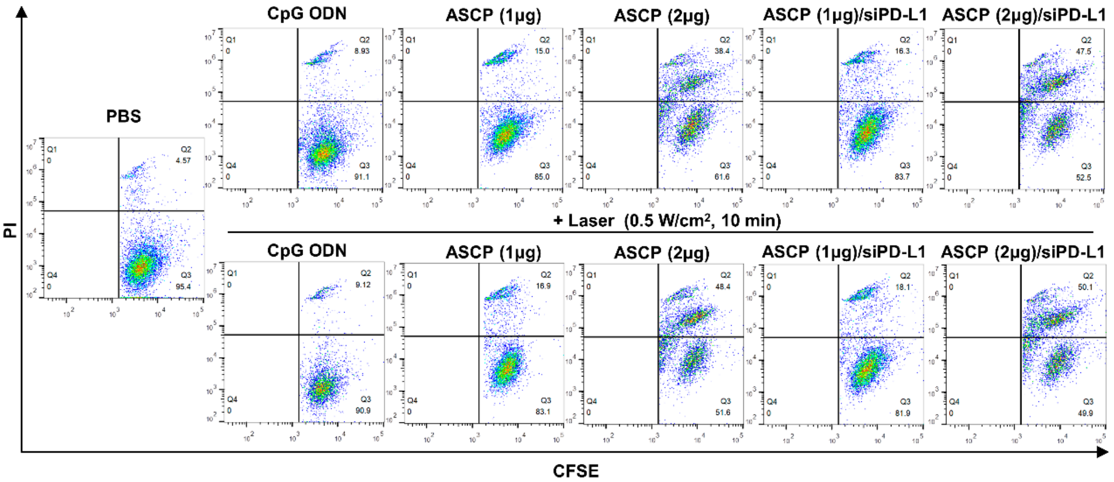

Figure S15. MC38 cells treated with different formulations of NPs with or without laser radiation were co-incubated with BMDCs and splenocytes for 48 h, then the killing

effect on MC38 cells was analyzed with flow cytometry by gating in $CFSE^{+}PI^{+}$. MC38 cells were pre-stained with CFSE, and the killed MC38 cells were detected by PI.

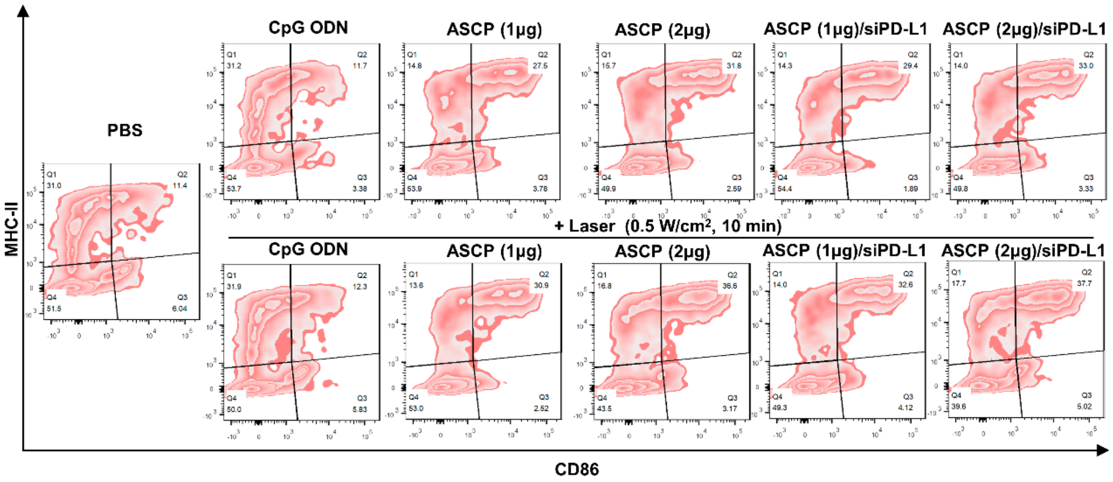

Figure S16. MC38 cells treated with different formulations of NPs with or without laser radiation were co-incubated with BMDCs and splenocytes for 48 h, then the maturity of BMDCs was analyzed by flow cytometry. CD86+MHC-II+ cells gated in CD11c+ cells were mature DCs.

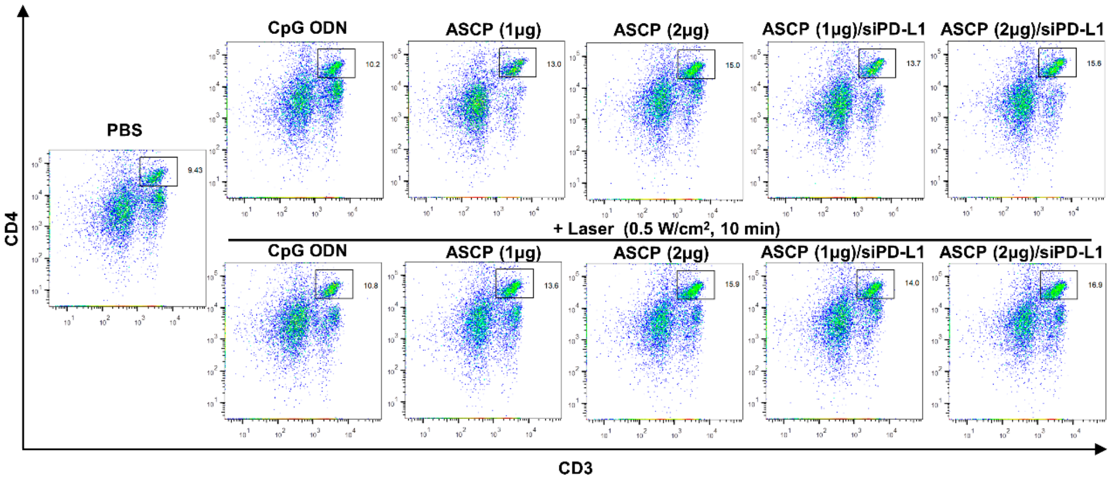

Figure S17. MC38 cells treated with different formulations of NPs with or without laser radiation were co-incubated with BMDCs and splenocytes for 48 h, then the differentiation of splenic lymphocytes was analyzed by flow cytometry. CD3 was the marker of T lymphocytes, and CD4 and CD8 were the markers of effector T lymphocytes.

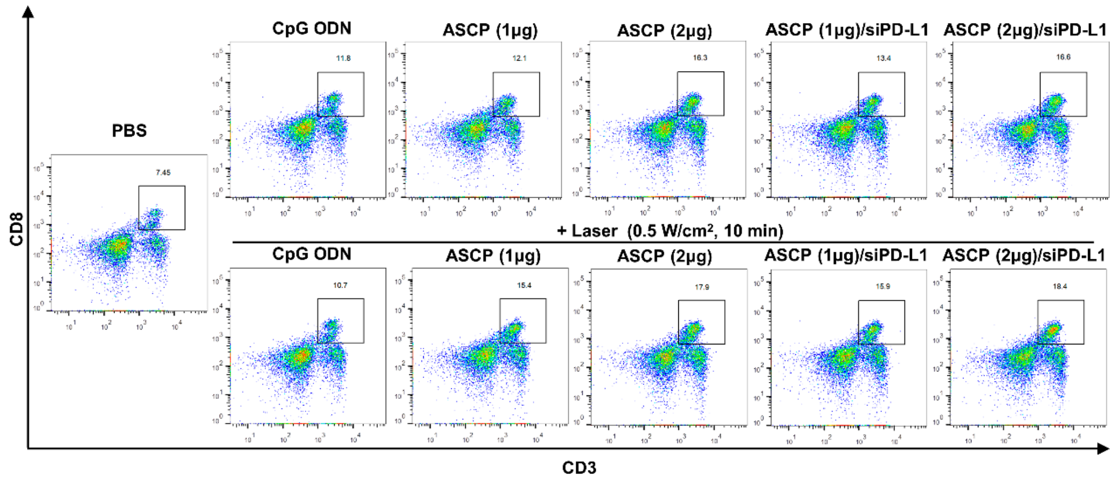

Figure S18. MC38 cells treated with different formulations of NPs with or without laser radiation were co-incubated with BMDCs and splenocytes for 48 h, then the differentiation of splenic lymphocytes was analyzed by flow cytometry. CD3 was the marker of T lymphocytes, and CD4 and CD8 were the markers of effector T lymphocytes.

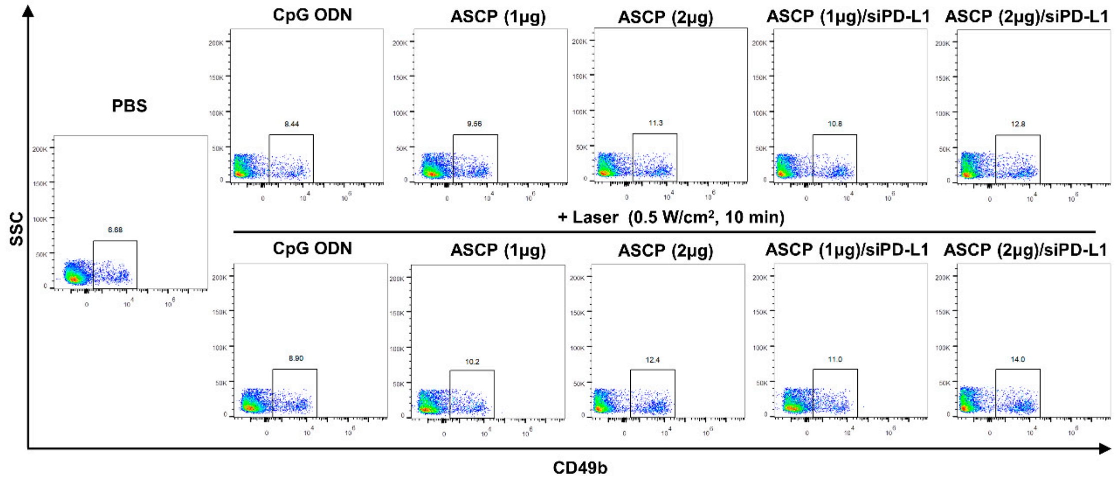

Figure S19. MC38 cells treated with different formulations of NPs with or without laser radiation were co-incubated with BMDCs and splenocytes for 48 h, then the differentiation of splenic lymphocytes was analyzed by flow cytometry. CD49b was the marker of NKs.
